# A Flow Cytometry-Based Phenotypic Screen To Identify Novel Endocytic Factors in *Saccharomyces cerevisiae*

**DOI:** 10.1534/g3.118.200102

**Published:** 2018-03-14

**Authors:** Kristie Wrasman, Salvatore L. Alioto, Yorke Zhang, Kyle Hoban, Marjon Khairy, Bruce L. Goode, Beverly Wendland

**Affiliations:** * Department of Biology, Johns Hopkins University Baltimore, MD 21218; † Department of Biology, Brandeis University, Waltham, MA 02453

**Keywords:** Arp2/3, actin, clathrin-mediated endocytosis, cargo sorting, forward genetic screen

## Abstract

Endocytosis is a fundamental process for internalizing material from the plasma membrane, including many transmembrane proteins that are selectively internalized depending on environmental conditions. In most cells, the main route of entry is clathrin-mediated endocytosis (CME), a process that involves the coordinated activity of over 60 proteins; however, there are likely as-yet unidentified proteins involved in cargo selection and/or regulation of endocytosis. We performed a mutagenic screen to identify novel endocytic genes in *Saccharomyces cerevisiae* expressing the methionine permease Mup1 tagged with pHluorin (pHl), a pH-sensitive GFP variant whose fluorescence is quenched upon delivery to the acidic vacuole lumen. We used fluorescence-activated cell sorting to isolate mutagenized cells with elevated fluorescence, resulting from failure to traffic Mup1-pHl cargo to the vacuole, and further assessed subcellular localization of Mup1-pHl to characterize the endocytic defects in 256 mutants. A subset of mutant strains was classified as having general endocytic defects based on mislocalization of additional cargo proteins. Within this group, we identified mutations in four genes encoding proteins with known roles in endocytosis: the endocytic coat components *SLA2*, *SLA1*, and *EDE1*, and the *ARP3* gene, whose product is involved in nucleating actin filaments to form branched networks. All four mutants demonstrated aberrant dynamics of the endocytic machinery at sites of CME; moreover, the *arp3^R346H^* mutation showed reduced actin nucleation activity *in vitro*. Finally, whole genome sequencing of two general endocytic mutants identified mutations in conserved genes not previously implicated in endocytosis, *KRE33* and *IQG1*, demonstrating that our screening approach can be used to identify new components involved in endocytosis.

Endocytosis is an essential process in eukaryotic cells that enables nutrient uptake, response to extracellular signals, and maintenance of proper protein and lipid composition at the plasma membrane (PM) . Although a variety of endocytic mechanisms exists (Kumari *et al.* 2010), clathrin-mediated endocytosis (CME) is the best-studied endocytic pathway and accounts for the majority of uptake from the PM in most cells (Bitsikas *et al.* 2014; Goode *et al.* 2015). During CME, cytosolic proteins are recruited to endocytic sites at the PM in a highly coordinated manner such that early-arriving proteins facilitate the recruitment of later-acting endocytic proteins, which in turn promote actin polymerization to drive membrane invagination and vesicle scission (Kaksonen *et al.* 2003; 2005; Taylor *et al.* 2011). The endocytic machinery assembles at discrete PM sites, and cells exhibit a characteristic number of endocytic patches with well-defined lifetimes and orders of recruitment for CME proteins at these sites (Kaksonen *et al.* 2003; 2005; Taylor *et al.* 2011). Importantly, the CME machinery is highly conserved through evolution, and studies in the budding yeast *Saccharomyces cerevisiae*, have first defined roles for many endocytic proteins that turn out to have similar functions in mammalian cells (Boettner *et al.* 2012).

In yeast, CME can be broken down into three major stages of protein recruitment to endocytic patches: the early coat phase, the late coat phase, and the actin polymerization/scission phase (Kaksonen *et al.* 2006). Interactions between cargo and the early coat proteins, including clathrin, endocytic adaptors and accessory proteins, likely establish the endocytic site and subsequently permit recruitment of late coat proteins as the site matures (Stimpson *et al.* 2009; Carroll *et al.* 2012; Suzuki *et al.* 2012; Lu and Drubin 2017). Many late coat proteins serve as a link between the early coat and actin polymerization (Newpher *et al.* 2005; Sun *et al.* 2006; Bradford *et al.* 2015). Proper formation of branched actin filament networks at sites of endocytosis requires the actin-nucleating Arp2/3 complex and other actin-binding proteins, including type I myosins. Together, these factors drive membrane internalization and scission of the vesicle (Sun *et al.* 2006; Barker *et al.* 2007). Overall, the genetic tools available in budding yeast provide many experimental advantages for studying endocytosis and continue to provide opportunities to further our understanding of the function and regulation of CME.

In response to changes in the external environment, a cell must selectively internalize specific proteins from the PM. Although ubiquitination plays a major role in directing internalization of specific cargos, the exact mechanisms controlling cargo selection during CME are not fully understood, and may involve additional proteins with no currently known roles in endocytosis (Hicke and Riezman 1996; Roth and Davis 1996). To address this, we designed a mutagenic screen to identify new endocytic, regulatory factors in budding yeast. Forward genetic screens have previously proven effective tools for characterization of complex cellular processes such as endocytosis. Earlier screening approaches to identify endocytic components have used either membrane reporters such as the lipophilic styryl dye FM4-64 combined with the enrichment for temperature-sensitive mutants, or have utilized previously identified endocytic proteins as partners for genetic or physical interactions with unknown factors (Raths 1993; Holtzman *et al.* 1993; Wendland *et al.* 1996; Boettner *et al.* 2009; Burston *et al.* 2009; Michelot *et al.* 2010; Farrell *et al.* 2015). In this study, we selected mutants based solely upon the phenotype of reduced ability to internalize an endogenous cargo and did not rely upon a secondary growth phenotype such as temperature sensitivity. We performed random mutagenesis of WT cells using ethyl methanesulfonate (EMS), which induces point mutations that transition G:C base pairs to A:T base pairs (Lee *et al.* 1990). This strategy allows unbiased identification of missense mutations and truncations in proteins, both of which can illuminate functional domains and important amino acid(s) necessary for the endocytic function of a given protein.

We examined the internalization of the endogenous endocytic cargo Mup1, a high-affinity methionine permease that undergoes nutrient-regulated endocytosis (Isnard *et al.* 1996). In methionine-limiting conditions, Mup1 is trafficked to and retained at the PM. Upon addition of excess methionine, Mup1 is rapidly trafficked to the vacuole for degradation (Lin *et al.* 2008; Prosser *et al.* 2010). Mup1 internalization can be monitored using a GFP tag; however, GFP is resistant to vacuolar proteolysis and accumulation of stable GFP in the vacuole confounds quantification of endocytic events (Bokman and Ward 1981; Prosser *et al.* 2010; Prosser *et al.* 2016). In contrast, superecliptic pHluorin (pHl) is a pH-sensitive variant of GFP that exhibits bright fluorescence at neutral pH, but this fluorescence is quenched when the protein is exposed to acidic environments such as the yeast vacuole lumen (Miesenböck *et al.* 1998; Sankaranarayanan *et al.* 2000; Prosser *et al.* 2010). By using a Mup1-pHl fusion construct, Mup1 internalization can be assessed by measuring fluorescence of whole cells before and after treatment with methionine using both fluorescence microscopy and flow cytometry. Because the C-terminal pHluorin tag is fused to the cytoplasmic tail of Mup1, cells are brightly fluorescent when Mup1 localizes to the PM or early endosomes, but are dim when Mup1 is incorporated into multivesicular bodies and subsequently transported to the vacuole (Prosser *et al.* 2010; Prosser *et al.* 2016). Thus, analysis of Mup1-pHl in mutagenized cells can allow detection of strains with mutations in endocytic genes, since a block or delay in transport of Mup1-pHl to the vacuole results in cells that remain fluorescent, in contrast to WT cells that rapidly lose fluorescence as Mup1 is internalized and targeted to the vacuole.

In performing an unbiased, forward genetic screen using cargo mislocalization as the sole phenotype, we anticipated that we would isolate novel mutations in genes with known roles in CME, as well as mutations in genes with no previously identified endocytic function. Accordingly, our screen identified truncations in the endocytic coat genes, *SLA2*, *SLA1*, and *EDE1*, as well as a novel missense mutation in the Arp2/3 complex subunit gene, *ARP3*. Additionally, we identified missense mutations in *KRE33* and *IQG1*, whose protein products are canonically involved in ribosome assembly (Kre33) and cell polarization and cytokinesis (Iqg1) (Machesky 1998; Li *et al.* 2009). Neither *KRE33* nor *IQG1* has been previously implicated in endocytosis, indicating that our screening approach can identify novel proteins with roles in CME.

## Materials and Methods

### Strains and Plasmids

A complete list of strains and plasmids used in this study can be found in Table S1 and Table S2, respectively. Strain construction via genomic integration using PCR-based methods was performed as previously described (Longtine *et al.* 1998). All cells were grown in either YPD or YNB medium lacking the appropriate amino acids and nutrients when necessary to maintain plasmids.

### Fluorescence Microscopy and Flow Cytometry

Strains expressing genomically-encoded Mup1-pHl were grown in YNB -Met overnight to accumulate Mup1-pHl at the PM. Cultures were diluted between 0.1-0.25 OD in YNB -Met and grown to mid-logarithmic phase adding methionine to a final concentration of 20 μg/ml. Images were captured before and 45 min after methionine addition. Fur4-GFP expression was induced via the *CUP1* promoter by addition of 0.1 mM cupric sulfate throughout the experiment, and cells were grown in YNB -ura overnight (Jones *et al*. 2012; Keener and Babst 2013). Cultures were then diluted to 0.25 OD in YNB -ura containing 0.1 mM cupric sulfate and grown to mid-logarithmic phase before addition of uracil to a final concentration of 20 μg/ml. Images were captured 1 h after uracil addition. All other strains were grown in YNB medium for microscopy.

Images were captured using an Axiovert 200 inverted microscope (Zeiss) equipped with a Sensicam (Cooke), an X-Cite 120 PC fluorescence illumination system, a 100x, 1.4NA Plan-Apochromat oil immersion objective or with a microscope (Marianas; 3i) equipped with EM cameras (Cascade II 512; Photometrics), a 488-nm diode laser, an α-Plan-Fluor 100× 1.45 NA objective lens (Carl Zeiss), and Slidebook 5 software. All images were captured at room temperature with cells in YNB medium, and images within an experiment were captured using identical exposure conditions. Image J 1.48v was used for post-acquisition image analysis, and maximum and minimum intensity values were applied to images equally to maintain relative fluorescence intensity between strains within an experiment. One-way ANOVA with Tukey’s test was used for statistical significance testing, and was performed using Prism software (GraphPad). An F test was used to verify that the variance of distributions were significantly different between strains and was performed using Prism software (GraphPad). Flow cytometry data were acquired on a FACS Canto (BD Biosciences), and cells were analyzed in YNB medium with appropriate amino acids as described previously (Prosser *et al.* 2016).

### EMS Mutagenesis

Mid-logarithmic phase WT Mup1-pHl cells were separated into two populations, and grown in YNB -Met. Control cells were washed, and resuspended in YNB-Met for 45 min, then methionine was added to a final concentration of 20 µg/ml for 45 min. Cells were then sorted using a FACS Vantage (BD Biosciences), and sorting gates were set such that fewer than 0.5% of cells were sorted.

In order to introduce mutations, cells were treated with 3% (v/v) ethyl methanesulfonate (EMS) for 1 h in 0.1 M sodium phosphate buffer, pH 7.0 at 30°. EMS was inactivated by the addition of 5% sodium thiosulfate. After mutagenesis, cells were washed twice with YNB -Met medium and were then recovered in YNB -Met for 45 min at 30°. Methionine was added to a final concentration of 20 μg/ml for 45 min, and cells were sorted with the same gating as the control cells. Sorted cells were subsequently plated onto YPD medium and grown at 30°.

### Whole-genome sequencing and analysis

For each strain sequenced, genomic DNA was isolated using a DNeasy Plant Kit (Qiagen) according to the manufacturer’s instructions. Purified DNA samples were prepared for Illumina sequencing according to the manufacturer’s instructions at the Genetic Resources Core Facility (Johns Hopkins Institute of Genetic Medicine). Sequences were aligned to the S288C reference genome (UCSC, SacCer_Apr2011/sacCer3). VCF tools (Danecek *et al.* 2011) were used to remove variants specific to the parental strain, and Variant Effect Predictor (McLaren *et al.* 2016) was used to determine the mutated genes and consequence (missense, nonsense, or insertion/deletion) of each mutation.

### Protein purification

Rabbit skeletal muscle actin (RMA) (Spudich and Watt 1971), pyrenyliodoacetamide-labeled (pyrene) actin (Pollard and Cooper 1984), Oregon Green (OG)-labeled actin (Kuhn and Pollard 2005), the VCA fragment of Las17 (Winter *et al.* 1999), wildtype *S. cerevisiae* Arp2/3 complex, and arp3^R346H^ *S. cerevisiae* Arp2/3 complex (Smith *et al.* 2013) were purified as described.

### Pyrene–actin assembly assays

To prepare monomeric actin, pyrene-labeled RMA and gel-filtered, unlabeled RMA were centrifuged in parallel for 1 h at 90,000 rpm in a TLA100 rotor (Beckman Coulter). The upper 75% of each supernatant was carefully removed, actin concentrations were redetermined, and labeled and unlabeled RMA were mixed at a 1:19 ratio. Assembly reactions were performed in a 60 μl final volume and contained final concentrations of 2 μM G-actin (5% pyrene labeled). The actin mixture was converted to Mg-ATP-actin 1 min before use, and then 42 μl of actin was mixed with 15 μl of proteins or control buffer, plus 3 μl of 20 initiation mix (40 mM MgCl2, 10 mM ATP, 1 M KCl) to initiate polymerization. Pyrene fluorescence was monitored at 365 nm excitation and 407 nm emission at 25° in a fluorescence spectrophotometer (Photon Technology International, Lawrenceville, NJ).

### Total internal reflection fluorescence (TIRF) microscopy

Glass coverslips (24 × 60 mm #1.5; Fisher Scientific) were sonicated for 1 h in 2% Micro-90 detergent, followed by 1 h sonication in 100% ethanol, then 30 min sonication in 0.1 M KOH and distilled, deionized water, respectively. Cleaned coverslips were stored in 100% ethanol before use. Prior to imaging, each coverslip was rinsed with ddH20, dried with N_2_, and coated by applying 120 µl of 2 mg/ml methoxy-poly(ethylene glycol) (mPEG)-silane MW 2,000 (Laysan Bio) and 4 µg/ml biotin-PEG-silane MW 3,400 (Laysan Bio) resuspended in 80% ethanol, pH 2.0. Coated coverslips were incubated for 16 h at 70°. Flow cells were assembled by rinsing PEG-coated coverslips with distilled, deionized water, drying with N_2_, and adhering to µ-Slide VI0.1 (0.1 mm × 17 mm × 1 mm) flow chambers (Ibidi) with double-sided tape (2.5 cm × 2 mm × 120 µm) sealed with five-minute epoxy resin (Devcon). Flow cells were incubated for 1 min with 1% BSA, then incubated for 1 min with TIRF buffer (10 mM imidazole pH 7.4, 50 mM KCl, 1 mM MgCl_2_, 1 mM EGTA, 0.2 mM ATP, 10 mM DTT, 15 mM glucose, and 0.25% methyl cellulose [4000 cP]). TIRF reactions were initiated by adding 1 µM actin (20% OG-labeled) to premixed actin-binding proteins. Reactions were introduced into the flow chamber, which was then mounted on the microscope stage for imaging. Time-lapse imaging was performed on a Nikon-Ti200 inverted microscope (Nikon Instruments) equipped with a 488 nm argon laser (150 mW; Melles Griot), a 60× Apo oil-immersion TIRF objective (NA 1.49; Nikon Instruments), and an EMCCD camera with a pixel size of 0.267 µm (Andor), and running NIS-Elements (Nikon Instruments). Focus was maintained by the Perfect Focus system (Nikon Instruments). Images were collected at 5 s intervals for 15 min. Minimal contrast enhancement or changes to the black level were applied to the entire stack to improve image quality for analysis and display. Branched nucleation events were quantified every 60 s for 10 min, beginning 3 min after the initiation of each reaction, for at least 12 fields of view from a minimum of two independent reactions.

### Data availability

The sequencing data can be accessed at NCBI SRA database using the SRA identifier SRP132904. Supplemental material available at Figshare: https://doi.org/10.25387/g3.5970910.

## Results

### Proof of principle: Mup1-pHluorin as a tool to identify endocytosis mutants

**Figure 1:**
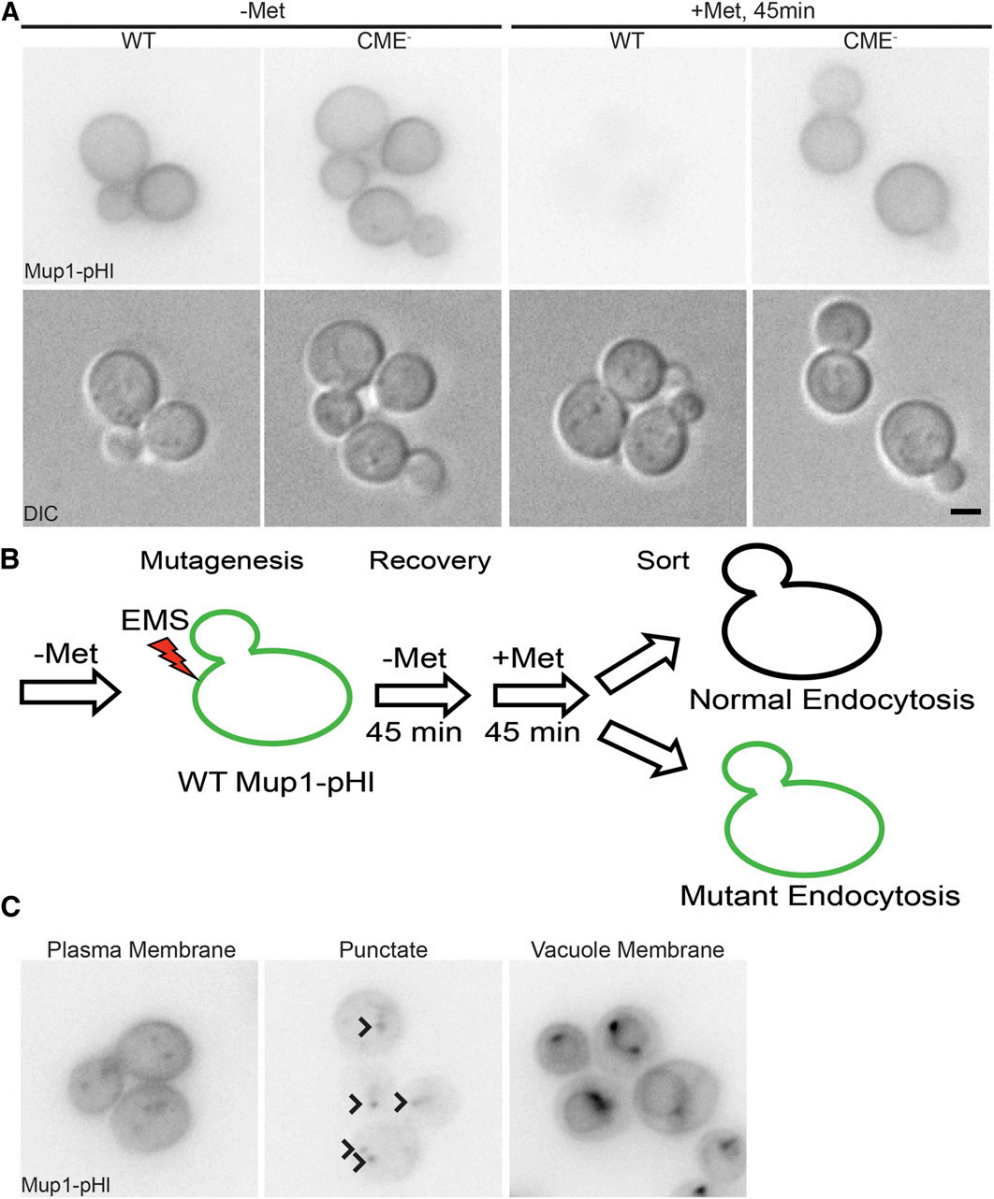
Endocytic function assayed during an EMS mutagenesis screen using an endogenous cargo, Mup1. A) Mup1-pHl localization was imaged using live-cell fluorescence microscopy (upper panel) and DIC optics (lower panel). WT and endocytic defective (CME^-^: *ent1Δ ent2Δ yap1801Δ yap1802Δ +ENTH1*) strains were imaged in medium lacking methionine (-Met) and 45 min after addition of 20 μg/ml of methionine (+Met) B) Schematic of mutagenesis screen workflow. C) Representative images of the three classes of mutants (Plasma membrane, Punctate, and Vacuole Membrane, as indicated) defined by Mup1-pHl localization 45 min after methionine addition. Arrowheads indicate punctae in the Punctate Class image. Scale bar, 2 μm.

To assess the feasibility of using Mup1-pHl as a tool for isolation of endocytosis mutants, we measured Mup1-pHl fluorescence intensity to establish differences between WT and endocytic defective cells. Yeast express four related endocytic adaptor proteins, Ent1, Ent2, Yap1801, and Yap1802, which together provide a direct link between the endocytic cargos to which they bind, PtdIns(4,5)P_2_ at the PM, and the clathrin coat (Wendland 1999; Kalthoff *et al.* 2002; Wendland 2002). Deleting both epsins (*ent1Δ ent2Δ*) is lethal; however, expression of the essential epsin N-terminal homology (ENTH) domain of either Ent1 or Ent2 is sufficient for cell viability (Wendland 1999). Although *ent1Δ ent2Δ + ENTH* cells have no discernable endocytic defect, simultaneous deletion of all four endocytic adaptors (*ent1Δ ent2Δ yap1801Δ yap1802Δ*) in cells expressing an ENTH domain produces a strain (henceforth referred to as CME^-^) that is viable but defective in endocytosis (Aguilar *et al.* 2006; Maldonado-Baez *et al.* 2008). In both WT and CME^-^ cells grown in medium lacking methionine, Mup1-pHl accumulated at the PM (Figure 1A). Forty-five minutes after addition of methionine, Mup1-pHl was barely detectable in WT cells due to cargo internalization and subsequent quenching of the pHluorin tag fluorescence in the vacuole (Prosser *et al.* 2010). As seen previously, CME^-^ cells were unable to efficiently internalize Mup1-pHl, and the cells remained fluorescent (Figure 1A) (Prosser *et al.* 2011; 2015).

In addition to visualizing Mup1-pHl by fluorescence microscopy, we compared whole-cell fluorescence intensity of WT and CME^-^ cells by flow cytometry, with the aim of establishing a method to enrich cells with endocytic defects. Using this approach, we observed an increase in fluorescence of CME^-^ cells compared to WT cells, consistent with retention of Mup1-pHl at the PM in CME^-^ cells (Figure S1). Specifically, we applied a vertical gate to the analyzed cells to distinguish between “bright” and “dim” fluorescence (to the right and to the left of the gate, respectively); in the experiment shown, only 7% of WT cells were categorized as bright, while 44% of CME^-^ cells were bright under identical gating conditions. As a proof of principle, we mixed a population of 90% WT and 10% CME^-^ cells, all expressing Mup1-pHl, treated them with methionine for 45 min, and enriched for bright cells using fluorescence-activated cell sorting. We then plated the collected cells, and the resultant colonies were categorized as WT or CME^-^ based on expression of distinct genetic markers. We obtained an enrichment of bright, CME^-^ cells, indicating that we could identify and isolate a minority of endocytic mutants from a larger population of WT cells (K. Wrasman and B. Wendland, unpublished data).

### A flow cytometry-based screen for cells with defective Mup1 internalization yielded three classes of endocytic mutants

Our ability to sort CME^-^ cells from their WT counterparts suggested that a genetic screen would similarly be able to isolate mutants defective in endocytosis. Thus, we mutagenized WT cells expressing Mup1-pHl with EMS to induce random point mutations (Figure 1B). A brief recovery time was chosen to allow the treated cells to express mutant alleles, (with some turnover of pre-existing wild type protein) while minimizing cell division that would lead to duplication of mutant cells. Methionine was then added, and bright cells were enriched using flow cytometry after a 45 min incubation to allow for Mup1-pHl internalization. A highly selective gate was applied to the control, non-mutagenized population of cells that sorted less than 0.5% of the population. Using an identical gate setting for the mutagenized cells, 6% of the total cell population was collected, indicative of an increased population of cells with defects in Mup1-pHl trafficking following EMS mutagenesis (Figure S2). After sorting, collected cells were plated on YPD rich medium, and viable cells formed 1525 individual colonies. We performed a secondary screen of 1003 colonies using fluorescence microscopy to assess Mup1-pHl localization. From these colonies, we confirmed endocytic or post-endocytic trafficking defects in 256 mutant strains, which were placed into three major classes based on the primary localization of Mup1-pHl after 45 min treatment with methionine: plasma membrane, punctate, and vacuolar membrane (Figure 1C, Table S3). The plasma membrane class (64% of the mutants) includes mutants with PM only and with PM and punctae localization of Mup1-pHl. These mutants may represent defects that either block or slow the endocytic process, since Mup1-pHl accumulation in internalized punctae indicates some residual endocytic function. The punctate class (31% of the mutants) likely represents defects in endosomal maturation and sorting. In the final class of mutants (5%), Mup1-pHl localized to the vacuole membrane, similar to the well-characterized *vps* class E phenotype of blocked cargo sorting into the luminal vesicles of multivesicular bodies (Raymond *et al.* 1992). In many previous genetic screens, temperature sensitivity was used as an additional criterion for mutant selection (Raths 1993; Wendland *et al.* 1996). For our mutants, only 19 of the 256 were heat-sensitive at 37°, and 8 were cold-sensitive at 18°. Thus, mutations identified in our screen may be unique and unrepresented in other screens.

Following confirmation of defective Mup1-pHl trafficking, we determined whether the isolated mutants were dominant or recessive using only the Mup1-pHl assay. Each haploid mutant (*MATα*) was mated with the haploid Mup1-pHl-expressing WT strain of the opposite mating type (*MAT****a***) to create a heterozygous strain. To our knowledge, the assay is fundamentally the same for both haploids and diploids. There was no difference seen in uptake of Mup1 in haploid or diploid WT cells at the 45 min time point. In each case, whole-cell Mup1-pHl fluorescence after the addition of methionine was assessed by flow cytometry for both the haploid and the heterozygous diploid strain. When the heterozygous diploid strain showed a decrease in fluorescence that was similar to WT, the mutant was classified as recessive, since the parental WT allele was sufficient to restore Mup1-pHl trafficking (Figure S3). In contrast, when the heterozygous diploid strain showed an elevated level of fluorescence similar to that of the corresponding mutant haploid strain, the mutant was classified as dominant. Based on these criteria, 135 mutants were categorized as dominant, and 118 mutants were categorized as recessive (Table S3). Two mutants were unable to mate and were not studied further. For the remainder of this study, we focused on the 47 recessive mutants with PM-localized Mup1-pHl.

### PM-class endocytic mutants showed differential internalization of four endocytic cargos

In order to determine whether the endocytic defects in the 47 recessive mutants were specific to Mup1 or applied more generally to other cargos, we monitored the trafficking of two other known endocytic cargo proteins: Snc1 and Ste3. Snc1 is a v-SNARE involved in secretory vesicle fusion at the PM and is normally trafficked to sites of polarized growth at the plasma membrane. Snc1 is then recycled via CME to permit further rounds of vesicle fusion (Gurunathan *et al.* 2000; Lewis *et al*. 2000). Under normal conditions, PM-localized Snc1 concentrates at the bud; however, when endocytosis is disrupted, GFP-Snc1 localization at the PM becomes depolarized to both the growing bud and mother cell (Valdez-Taubas and Pelham 2003). Ste3 is a G protein-coupled receptor (GPCR) for the **a**-factor mating pheromone that is constitutively trafficked to the plasma membrane, internalized, and targeted to the vacuole (Urbanowski and Piper 2001). In cells with endocytic defects, Ste3-GFP shows a stronger retention at the PM compared to WT cells in which Ste3-GFP localization is predominantly vacuolar (Chen and Davis 2000). Thus, steady-state localization of both GFP-Snc1 and Ste3-GFP can be used to detect defects in endocytosis for these constitutively-internalized cargos.

**Figure 2:**
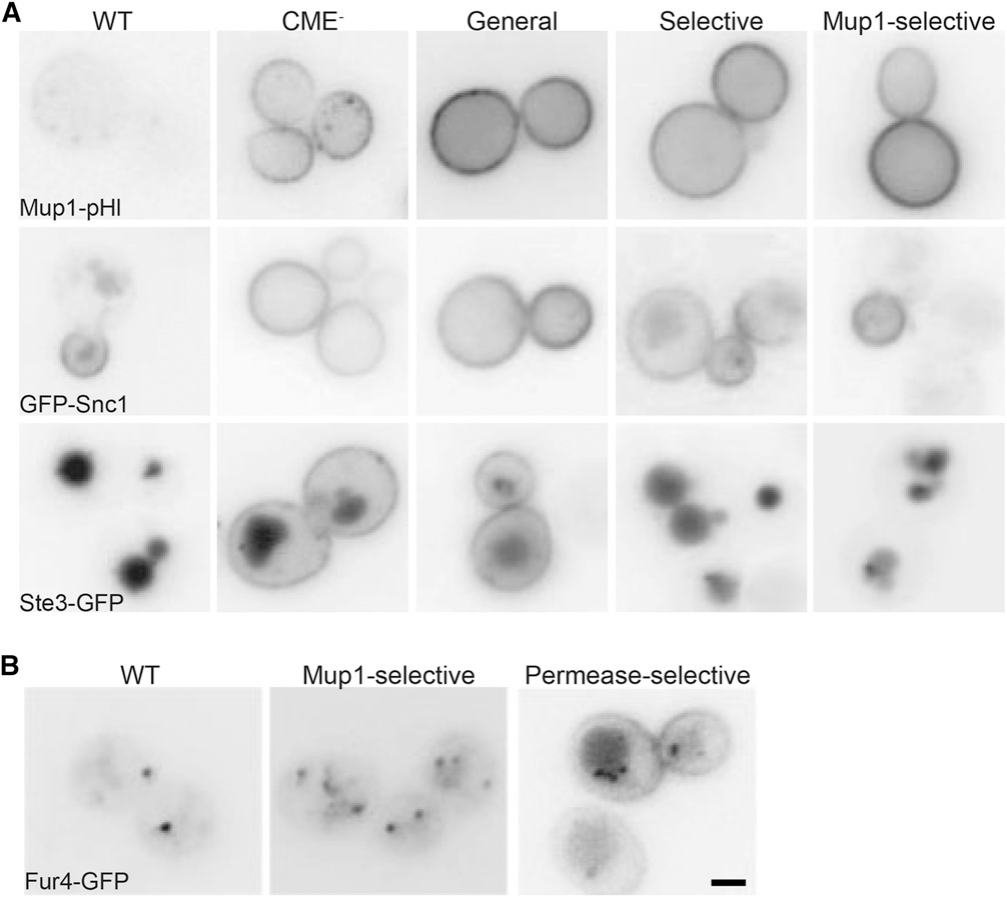
Subclassification of the Plasma Membrane class mutants based on differential internalization of endocytic cargos. A) WT, CME^-^, and mutant strains representing general, selective, and Mup1-selective subclasses of mutants were imaged via fluorescence microscopy to observe differences in localization of fluorescently-tagged endocytic cargoes, Mup1-pHl (top row), GFP-Snc1 (middle row), and Ste3-GFP (bottom row). Mup1-pHl was imaged by live-cell microscopy after a 45 min methionine treatment. GFP-Snc1, and Ste3-GFP were expressed from low-copy plasmids. B) Fur4-GFP expressed in WT and mutant strains representing Mup1-selective and permease-selective subclasses were grown in YNB medium lacking uracil. 20 μg/ml of uracil was added for 1 h prior to to induce internalization of Fur4-GFP for imaging by live-cell microscopy. Scale bar, 2 μm.

Of the 47 recessive, PM-class mutants analyzed, 15 showed a disruption in endocytosis of both GFP-Snc1 and Ste3-GFP, in addition to Mup1-pHl, and were thus characterized as general endocytic mutants (Figure 2A, Table S3). Thirteen mutants exhibited an unexpected endocytic defect in which endocytosis of Mup1, along with either Snc1 or Ste3, but not both, was disrupted (Figure 2A). Selective cargo effects like these have not been observed previously to our knowledge, making these unique mutants excellent subjects of future study. The remaining 19 mutants showed only retention of Mup1 at the PM and were characterized as Mup1-selective. Because both Snc1 and Ste3 are constitutively internalized, we tested whether the Mup1-selective subclass could still internalize the uracil permease Fur4, another cargo that undergoes nutrient-regulated endocytosis (Blondel *et al.* 2004). This assay identified an additional subclass of eight permease-selective mutants in which internalization of both Mup1 and Fur4 was defective, leaving eleven mutants that were exclusively deficient in trafficking Mup1 (Figure 2B, Table S3). While the general endocytic mutants will likely identify core proteins that mediate CME, future identification and characterization of mutants in the other subclasses may be of great value in identifying the factors that contribute to cargo selectivity and/or environmental responsiveness.

### The α-arrestin LDB19/ART1 restores Mup1-pHluorin endocytosis in seven mutant strains

Three factors implicated in Mup1-selective internalization include the α-arrestin Ldb19, the ubiquitin ligase Rsp5, and the protein kinase Npr1 (Lin *et al.* 2008; MacGurn *et al.* 2011). We expressed each gene from a low-copy plasmid and assessed complementation of the mutant phenotype using the Mup1-pHl localization microscopy assay (K. Wrasman, M. Khairy and B. Wendland, unpublished data). When a plasmid containing an endocytic gene complemented a mutant strain, the genomic copy from the mutant strain was amplified and sequenced. *LDB19* expressed from a low-copy plasmid complemented the Mup1 endocytic defect in 7 of 19 mutants within either the Mup1-selective (three mutants) or permease-selective (four mutants) classes (Figure S4). Ldb19 promotes the internalization of specific cargo proteins by recruiting the Rsp5 ubiquitin ligase for both Mup1 and Fur4 (Lin *et al.* 2008; Nikko and Pelham 2009). However, when we sequenced the *LDB19* locus (including approximately 500 bp upstream and downstream of the coding region) in each of these seven strains, we found no mutations relative to the wild type gene. The lack of mutations in either the coding sequence or the typical regulatory regions suggest that increased *LDB19* copy number is likely acting as a suppressor of the Mup1-pHl internalization defect in these mutants.

Many cargo proteins are ubiquitinated to promote their internalization and subsequent degradation in the vacuole (Gagny *et al.* 2000; Kalthoff *et al.* 2002; Shih *et al.* 2002; Blondel *et al.* 2004; Lin *et al.* 2008). Mup1 and Fur4 are both ubiquitinated by Rsp5 to induce internalization, whereas Npr1 phosphorylation stabilizes transporters at the PM (Kaouass *et al.* 1998; De Craene *et al.* 2001; Lin *et al.* 2008; MacGurn *et al.* 2011). Expressing *RSP5* and *NPR1* from a plasmid did not complement the endocytic phenotype in any of the 47 mutants tested; this is perhaps not surprising since both Rsp5 and Npr1 have multiple cellular roles in addition to their function in endocytosis.

### Identification of causative mutations

**Figure 3:**
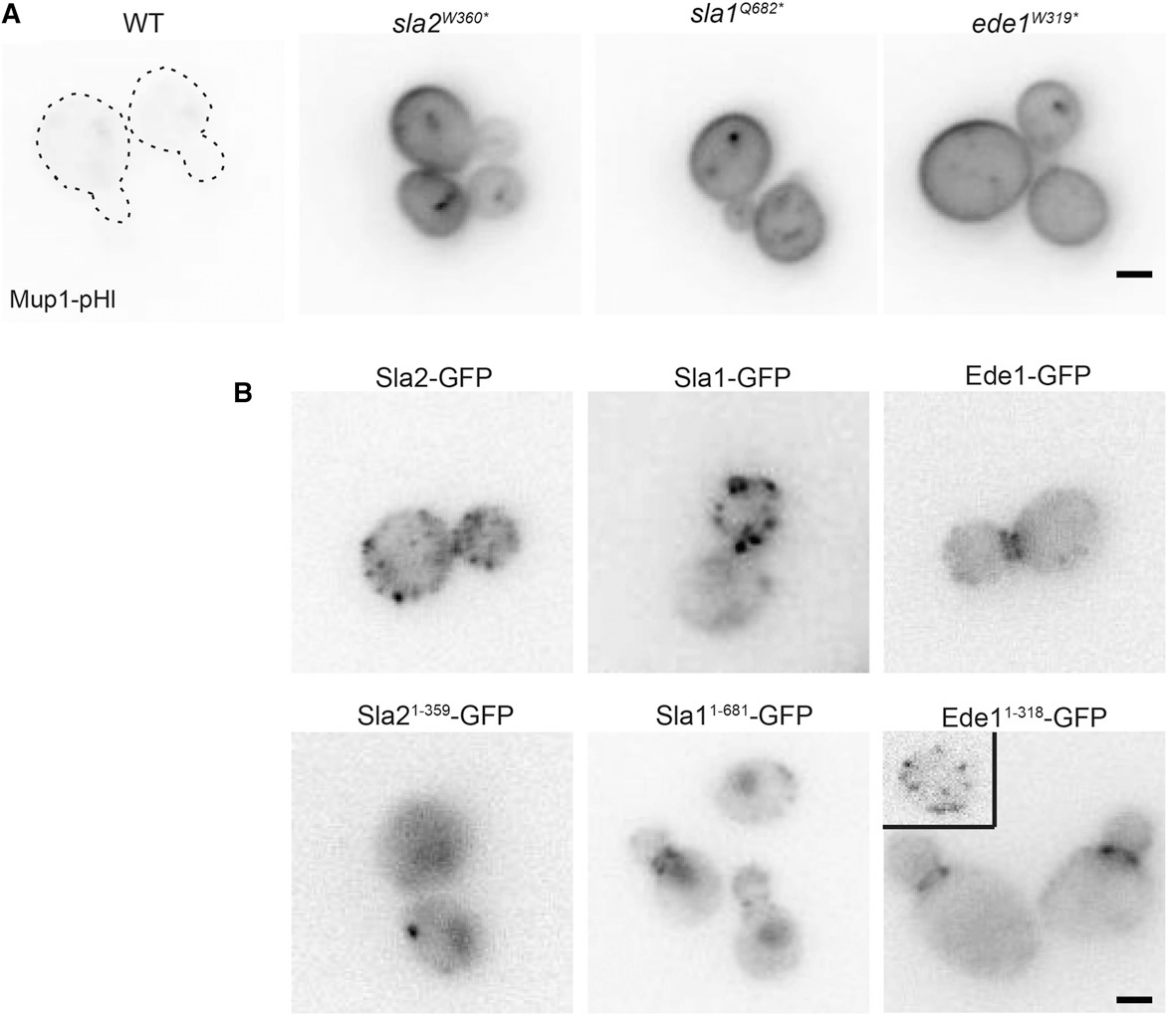
*SLA2*, *SLA1*, and *EDE1* truncations cause defects in endocytosis A) In a WT strain expressing Mup1-pHl, the specified amino acid in *SLA2*, *SLA1*, or *EDE1* was mutated to a stop codon, generating the truncations. Mup1-pHl localization was assessed by fluorescence microscopy 45 min after addition of methionine. Dashed lines indicate the plasma membrane of WT Mup1-pHl cells. B) Strains expressing full-length and truncated Sla2, Sla1, and Ede1 were generated by the integration of GFP tag at the endogenous locus and imaged by fluorescence microscopy. Scale bar, 2μm.

Mutations in many proteins are known to disrupt endocytosis. Thus, we chose 13 additional candidate genes involved in CME to test for complementation of our 47 endocytic mutants (Table S4). These 13 genes represent components of the endocytic machinery that act in all stages of a CME event and result in endocytic defects when individually mutated or deleted. When expressed from low-copy plasmids, *SLA2* and *SLA1* complemented the Mup1 endocytic defect, and we identified mutations in these two CME machinery genes: nonsense mutations in *SLA2* at amino acid 360 (sla2^W360*^) and in *SLA1* at amino acid 682 (sla1^Q682*^) (Figure 3A, Figure S5C, Table S3). The remaining tested mutants were not complemented by expression of any candidate genes.

We used whole-genome sequencing to identify the causative, EMS-induced point mutations for the endocytic defect of five general endocytic defective mutant strains that were not complemented by plasmids encoding candidate proteins. To reduce the number of irrelevant mutations in the genome, these strains were backcrossed multiple times to the non-mutagenized parent strain. The mutants and the parent strain were sequenced, and all mutations identified in the parent strain were removed to generate a set of candidate causative mutations for the endocytic defects. The final list of candidate mutations was generated to include mutations in coding sequences that caused a missense or nonsense mutation. Although this technique is highly sensitive, it was not optimal for all the mutants sequenced. For example, some mutant strains were unable to sporulate, and therefore, could not be backcrossed to the parent strain; sequencing results from two of these strains identified hundreds of point mutations in each instead of the 40 or fewer mutations that were typically found in backcrossed strains. Of the strains analyzed by whole-genome sequencing, one mutant contained the same sla1^Q682*^ nonsense allele that was discovered via our candidate approach; this mutant had not been tested for complementation with *SLA1* prior to sequencing. Another strain contained a mutation in the CME machinery gene *EDE1* that truncates the protein product at amino acid 319 (ede1^W319*^) (Figure 3A, Figure S5C). Finally, we identified missense mutations in *ARP3*, *KRE33*, and *IQG1* in three other mutant strains (see below).

### Functional analysis of truncation mutants in SLA2, SLA1, and EDE1

In order to confirm that the observed truncations in *SLA2*, *SLA1*, and *EDE1* caused the endocytic defects seen in the EMS-mutagenized strains, we recreated the truncated sla2^W360*^, sla1^Q682*^, and ede1^W319*^ alleles at the corresponding loci in the parental strain. Consistent with a role in endocytosis, truncation of these proteins caused a defect in Mup1 internalization as seen by retention of Mup1-pHl at the PM (Figure 3A). Next, we examined the localization of each truncated protein by generating GFP-tagged chimeras of the WT and truncated alleles via genomic integration. As reported previously, all three of the WT proteins localized to patches at the cell cortex (Figure 3B; Kaksonen *et al.* 2005). Prior studies have used an engineered sla2^W360*^ allele (Wesp *et al.* 1997; Yang *et al.* 1999; Sun *et al.* 2007); consistent with these previous studies in which truncated Sla2 is inefficiently recruited to the PM, Sla2^1-359^-GFP showed higher levels of cytoplasmic fluorescence with fewer Sla2^1-359^-GFP cortrical patches compared to the co-expressed CME site marker, Abp1-RFP (Figures 3B and Figure S5B). Similar to *sla2* mutants, many alleles of *SLA1* have been studied, and several truncated forms of Sla1 have been found to localize to the nucleus and to endocytic patches (Gardiner *et al.* 2007; Chi *et al.* 2012; Sun *et al.* 2015). Consistent with these findings, Sla1^1-681^-GFP localized to the nucleus and endocytic patches, though fluorescence intensity of this Sla1 truncation was diminished compared to those in WT patches (Figure 3B). Finally, Ede1^1-318^-GFP was observed primarily at patches near the bud neck, but it also localized faintly along the plasma membrane in a manner that was difficult to observe compared to the brighter bud neck localization (Figure 3B). The inset shows a more typical patch localization of Ede1 in an unbudded cell. Together, these data are consistent with mislocalization of the truncated proteins contributing to a disruption in the endocytic process.

### Sla2^W360*^, Sla1^Q682*^, and Ede1^W319*^ truncations alter protein dynamics at endocytic patches

**Figure 4:**
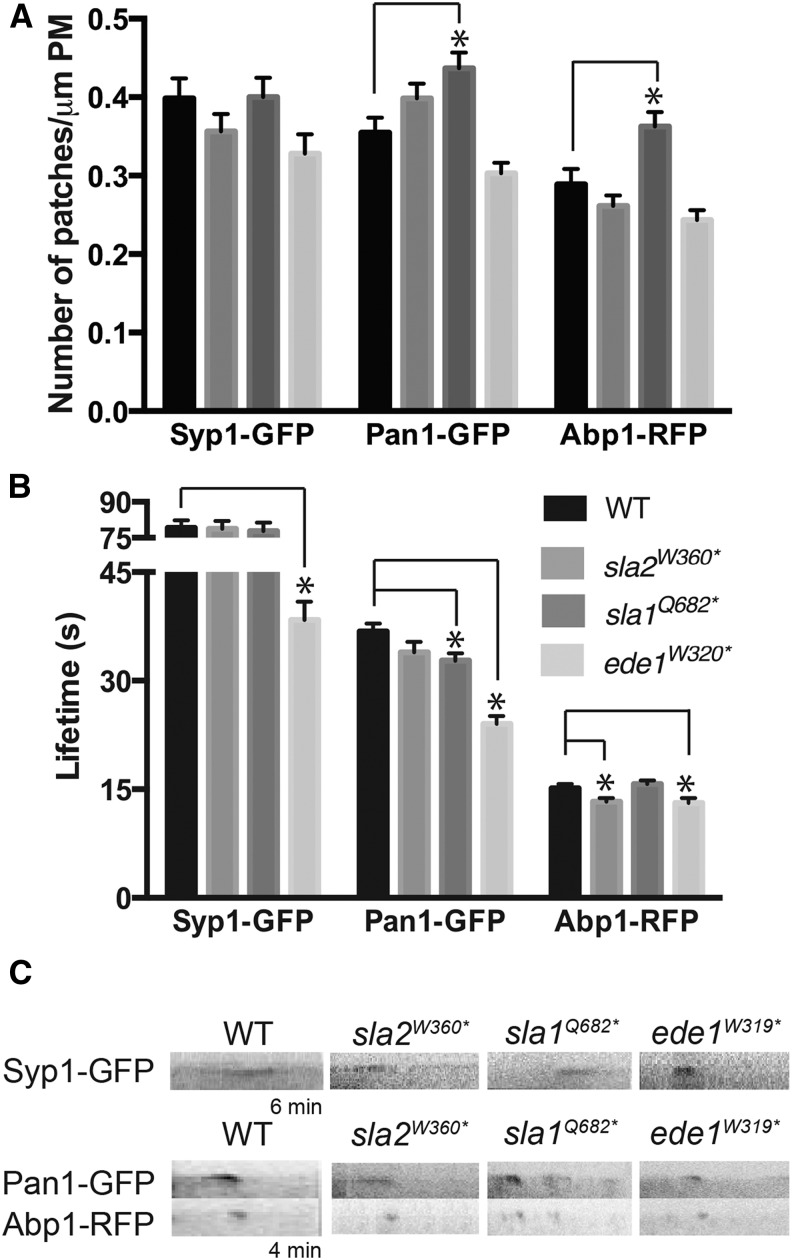
Effect of *SLA2*, *SLA1*, and *EDE1* truncation on endocytic patch number and lifetime of endocytic machinery proteins. A) Images of Syp1-GFP, Pan1-GFP and Abp1-RFP were acquired by live-cell time-lapse fluorescence microscopy, and the number of patches per micrometer of plasma membrane was counted for an equatorial section (N > 30 cells/strain). B) Lifetimes of Syp1-GFP, Pan1-GFP and Abp1-RFP calculated from movies of 120 frames acquired at intervals of 3 s (Syp1- GFP) or 2 s (Pan1-GFP, Abp1-RFP) (N > 50 patches/strain). All values from panels A and B are presented as mean ± SEM, * *P* < 0.05. C) Representative kymographs of Syp1-GFP, Pan1-GFP and Abp1-RFP in WT, sla2^W360*^, sla1^Q682*^and ede1^W319*^ strains. Kymographs were made from movies used for the quantification, and are oriented with cell interior at the bottom.

WT yeast cells have a characteristic number of patches, and lifetimes of proteins at endocytic sites are well defined (Kaksonen *et al.* 2003; 2005). CME has three major stages of protein recruitment to endocytic patches: early coat, late coat, and the actin phase, which correspond approximately to initiation (early) and maturation (late) of the endocytic site, followed by vesicle budding and scission (actin) (Goode *et al.* 2015). In order to determine which endocytic phase was disrupted by each truncation strain, we examined the number of endocytic patches and lifetimes of three proteins involved in CME: Syp1, Pan1, and Abp1, which are markers of the early coat, late coat, and actin nucleation phase, respectively. In all mutant strains, the number of Syp1-containing patches was indistinguishable from that observed in WT cells, suggesting that endocytic initiation is not perturbed by these truncations (Figure 4A and Figure S5A). Similarly, the sla2^W360*^ and ede1^W319*^ alleles did not affect the number of Pan1- or Abp1-containing patches at the cell surface. In contrast, sla1^Q682*^ had a significantly greater number of Pan1-GFP and Abp1-RFP patches compared to WT cells, suggesting that this truncation specifically affects the late coat machinery and endocytic site maturation.

Similar to phenotypes previously seen in *ede1*∆ cells, ede1^W319*^ cells showed a significant decrease in Syp1-GFP and Pan1-GFP lifetime at endocytic patches as well as a decrease in Abp1-RFP lifetime (Figure 4B, 4C; Reider *et al.* 2009; Stimpson *et al.* 2009). The ede1^W319*^ strain exhibited a disrupted timing of endocytic initiation, causing defects throughout the endocytic process; whereas both sla2^W360*^ and sla1^Q682*^ strains have normal Syp1-GFP lifetimes suggesting endocytic initiation was normal. sla2^W360*^ cells showed a significant decrease in lifetime of Abp1-RFP at endocytic sites, which was expected because the Sla2 C-terminus contains a domain that binds F-actin and contributes to the progression of endocytic events in the actin phase (Wesp *et al.* 1997; Yang *et al.* 1999; Boettner *et al.* 2009). The sla1^Q682*^ allele showed a slight decrease in Pan1-GFP lifetime at the endocytic patch but no change in the lifetime of Abp1-RFP, implying a mild maturation defect in CME (Figure 4B, 4C, Figure S5B). Overall, these data demonstrate that the truncation alleles of *SLA2*, *SLA1* and *EDE1* isolated in our screen perturb the dynamics of CME machinery proteins, which likely contributes to the observed delay in cargo internalization.

### An R346H point mutation in *ARP3* causes endocytic defects

**Figure 5:**
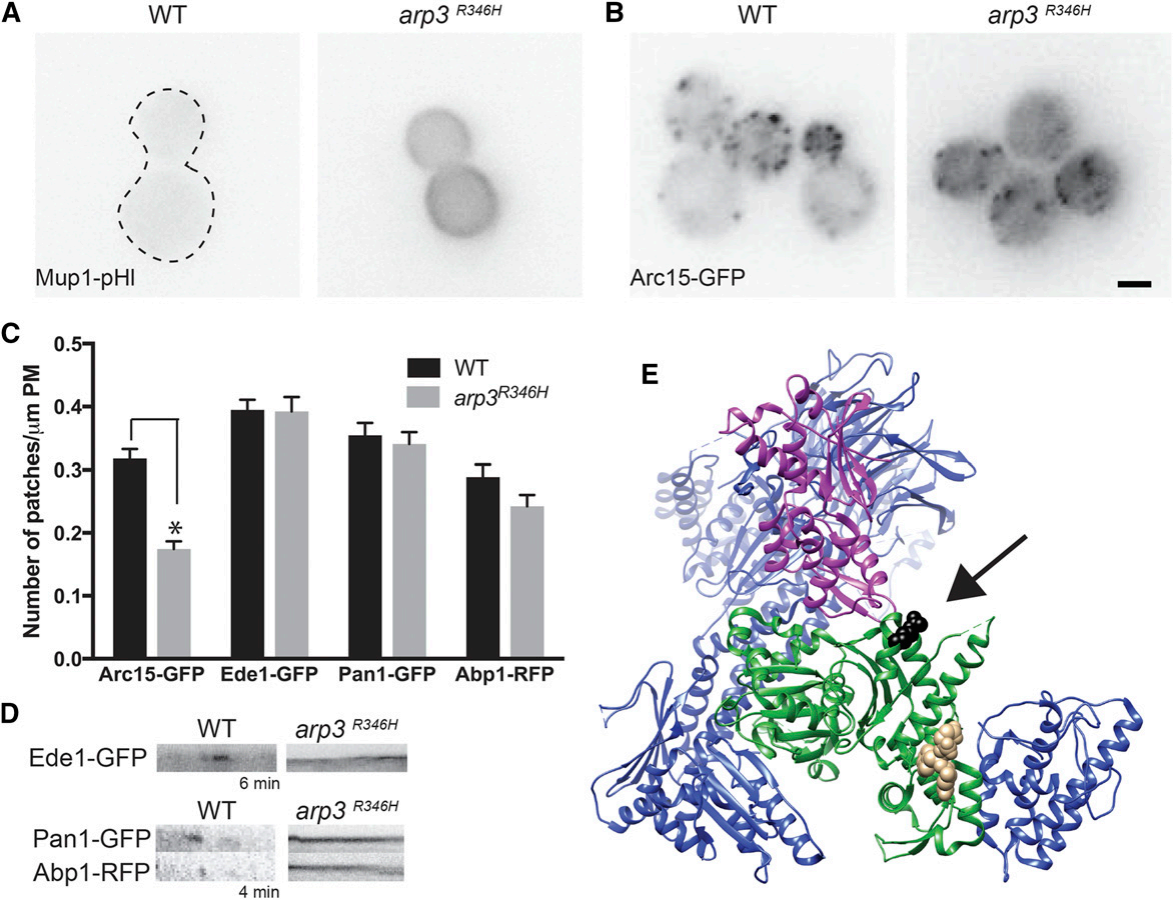
A missense mutation in *ARP3* causes endocytic defects A) In WT and *arp3^R346H^* expressing Mup1-pHl, cells were imaged by live cell microscopy 45 min after the addition of 20 μg/ml methionine. A dashed line indicates the plasma membrane of WT Mup1-pHl expressing cells. B) Images of Arc15-GFP in WT and *arp3^R346H^* cells were acquired by live-cell fluorescence microscopy. Scale bar, 2μm C) Time-lapse imaging of WT and *arp3^R346H^* cells expressing Arc15-GFP, Ede1-GFP, Pan1-GFP and Abp1-RFP were acquired, and the number of patches were per micrometer of plasma membrane was counted for an equatorial sections (N > 30 cells/strain). All values are presented as mean ± SEM, * *P* < 0.05. D) Representative kymographs of Ede1-GFP, Pan1-GFP and Abp1-RFP in WT, and *arp3^R346H^* strains. Kymographs were made from movies used for the quantification, and are oriented with cell interior at the bottom. E) Rendering was performed with Chimera (Pettersen *et al.* 2004) using the PDB file 3RSE of the bovine Arp2/3 structure (Ti *et al.* 2011). Arp3 is shown in green, Arp2 in purple, VCA in tan and the rest of the Arp2/3 complex members in blue. The amino acid in bovine Arp3 (R312) that corresponds to Arp3 (R346) in yeast shown in black.

Using whole genome sequencing, we identified an R346H mutation in the *ARP3* gene of one of the general endocytic mutant strains. To confirm that this mutation caused the observed endocytic defect, we independently generated the *arp3^R346H^* allele in the non-mutagenized, parental strain expressing Mup1-pHl and observed an endocytic defect similar to that seen in the original mutant (Figure 5A). Arp3 is one of the actin-related protein components of the Arp2/3 complex. Fluorescently-tagged Arp3 is not tolerated in yeast cells, and thus, a GFP chimera could not be generated (Kaksonen *et al.* 2005). Instead, we monitored localization of the Arp2/3 complex with Arc15-GFP, a subunit of the complex that is amenable to fluorescent tagging (Kaksonen *et al.* 2003). In *arp3^R346H^* cells, Arc15-GFP localized to fewer patches at the plasma membrane compared to WT cells and showed increased cytoplasmic localization (Figures 5, B and C). Early coat (Ede1-GFP), late coat (Pan1-GFP), and actin phase (Abp1-RFP) proteins each localized normally to endocytic sites in *arp3^R346H^* cells, and the patch number for these proteins was not significantly different from that of WT cells, unlike the reduction in Arc15-GFP patches (Figure 5C, Figure S6). However, Ede1-GFP, Pan1-GFP, and Abp1-RFP patches were stable throughout the period of data collection (4-6 min; Figure 5D). Similar to cells treated with the actin-depolymerizing drug Latrunculin A (LatA), progression of endocytic events in the *arp3^R346H^* strain was stalled, and the early and late coat proteins remained stable at cortical patches (Ayscough *et al.* 1997; Kaksonen *et al.* 2003; 2005). These data can be interpreted as endocytic machinery arriving at endocytic sites normally but being unable to complete vesicle formation.

To determine the mechanism of *arp3^R346H^* disruption of endocytosis, we modeled the mutation on a bovine Arp3 crystal structure obtained by crystallization of the intact Arp2/3 complex (Ti *et al.* 2011). The yeast Arg 346 residue aligns with the bovine Arg 312 residue, which is predicted to reside at the barbed end of Arp3 from which a daughter filament is nucleated and polymerization occurs to form a branch (Figure 5E). Comparing the sequences of *ARP3* and *ACT1*, it has been previously demonstrated that cells are inviable when the corresponding R290 amino acid in *ACT1* is mutated to alanine (along with the nearby K291, E292 residues) (DiPrima *et al.* 2014). Likewise, cells expressing *arp3^R346A^* were inviable; however, cells bearing the more conservative *arp3^R346K^* mutation were viable and showed no defects in Mup1-pHl endocytosis (K. Wrasman and B. Wendland, unpublished data).

**Figure 6:**
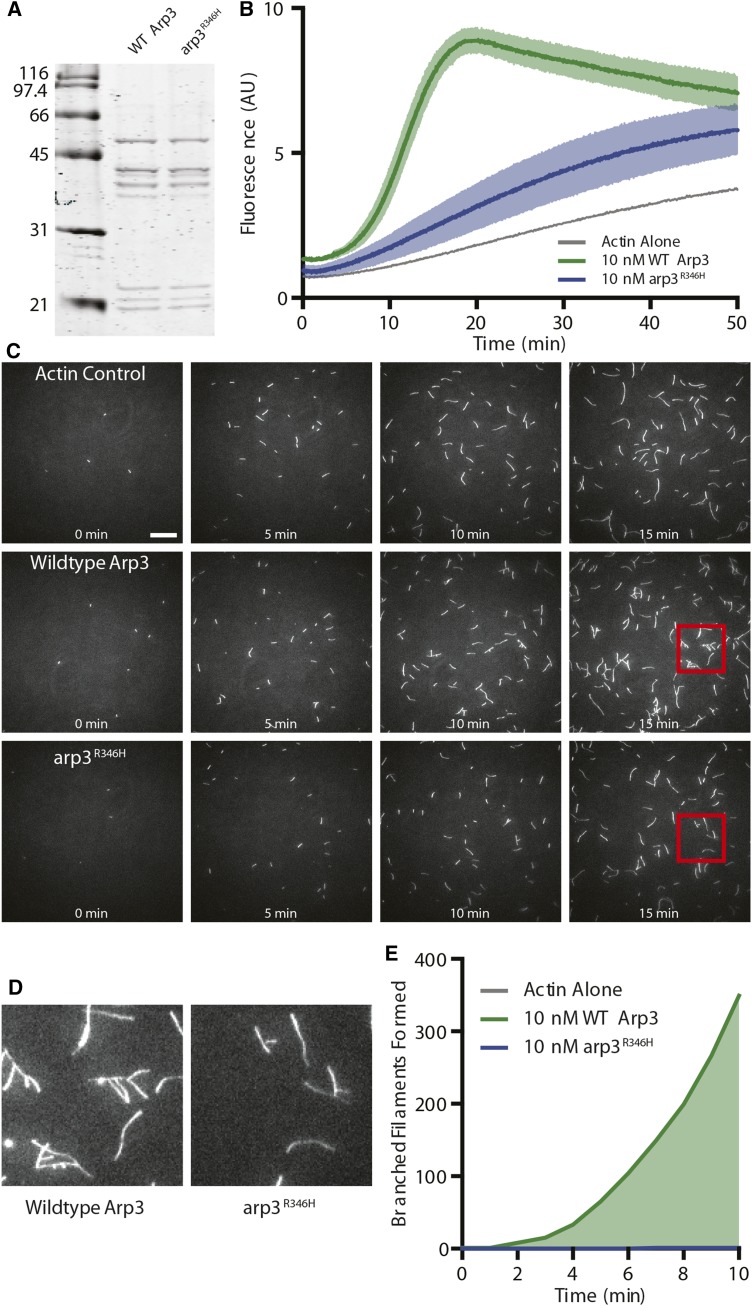
Biochemical characterization of Arp2/3 complex *vs.* Arp2/Arp3^R346H^ A) Side-by-side comparison of WT *vs.* Arp3^R346^*^H^* complex purified *in vitro*. B) Bulk pyrene-actin assembly assay comparing nucleation activity of Arp complex with WT *vs.* Arp3^R346H^ with Las17 VCA. Data averaged from n = 3 independent experiments. C) Representative time-lapse images from TIRF experiments containing 0.5 μM actin (10% OG-labeled) and 10 nM Arp2/3 complex containing wildtype or *arp3^R346H^* as indicated. Experiments containing Arp2/3 complex also contain 100 nM GST-Las17 VCA to stimulate Arp2/3 activation. Scale bar, 20μm. D) Magnified view of the regions boxed in red, indicated in C. E) Quantification of the number of branched filaments nucleated in the same reactions as D. Data combined from 6 fields of view per experiment, with 2-3 independent experiments (12-18 fields of view, total). Note, at 7 min *arp3^R346H^* nucleated a single, persistent branched actin filament. All values are presented as mean ± SEM.

To characterize the defect arising from the *arp3^R346H^* mutation, we purified WT Arp2/3 complex and mutant Arp2/3 complex carrying Arp3^R346H^ (Figure 6A) and compared their nucleation activities in pyrene-actin assembly assays. This kinetic assay measures the ability of Arp2/3 complex to nucleate actin polymerization, as pyrene-labeled actin shows increased fluorescence upon incorporation into filaments (Pollard and Cooper 1984). The mutant Arp2/Arp3^R346H^ complex showed a significant reduction in actin nucleation activity compared to WT Arp2/3 complex (Figure 6B).

A TIRF microscopy assay was used to directly visualize actin filament nucleation, extension, and branching. Reactions containing Arp2/Arp3^R346H^ had fewer total filaments compared to either WT Arp2/3 complex or actin alone (Figure 6C). This confirms the reduced polymerization activity seen in the pyrene assay. Only a single branched filament was observed in the TIRF reactions containing Arp2/Arp3^R346H^, in contrast to robust actin branching in reactions containing WT Arp2/3 complex (Figure 6C-E). These data suggest that the mutant Arp2/Arp3^R346H^ complexes nucleate actin at a drastically reduced level compared to WT Arp2/3 complex, explaining why few branches are seen in the TIRF assay.

### Identification of two point mutations suggests novel endocytic roles for KRE33 and IQG1

**Figure 7:**
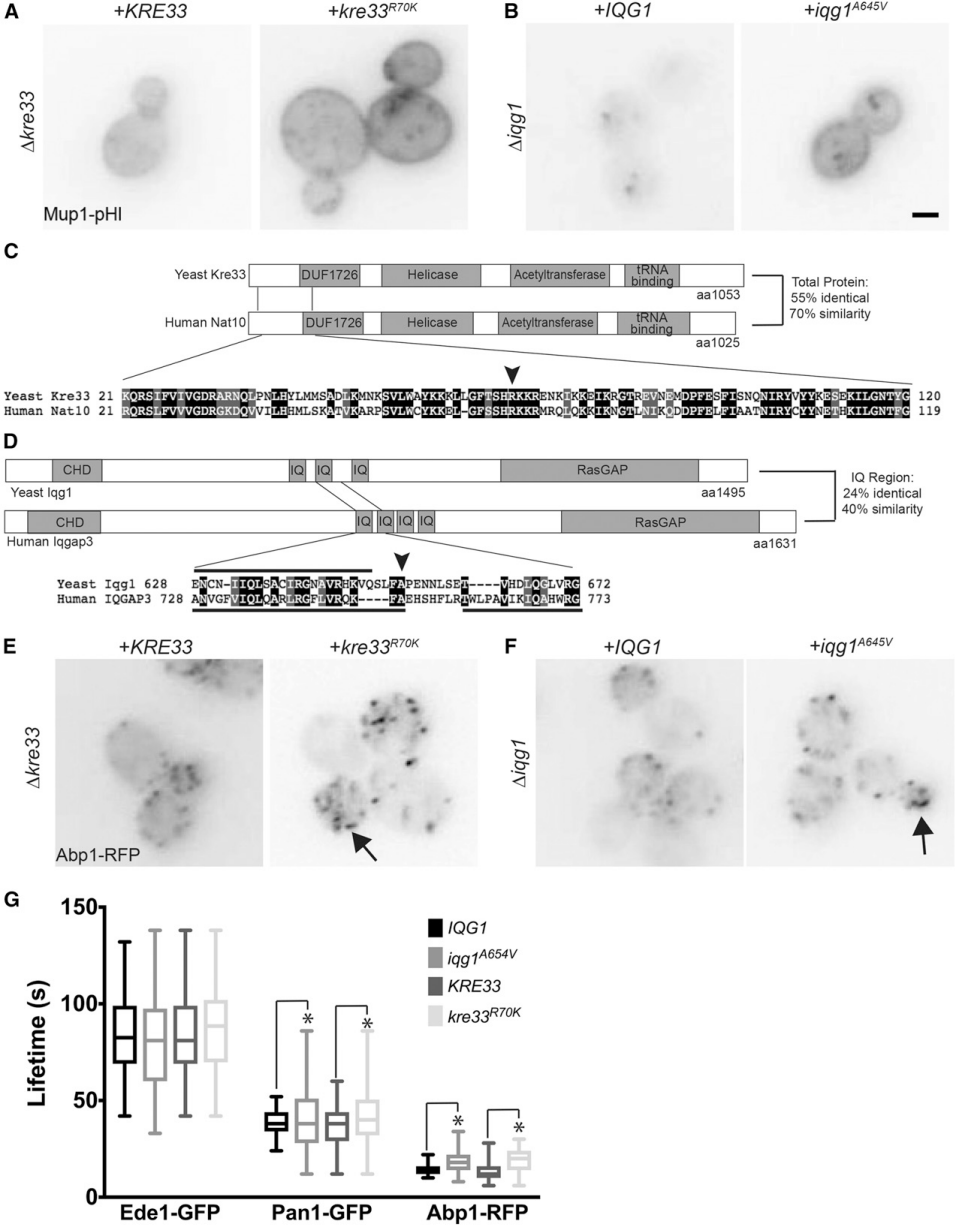
Effect of *kre33^R70K^* and *iqg1^A654V^* mutations on Mup1-pHl internalization A) In *kre33Δ* cells expressing Mup1-pHl, either *KRE33* or *kre33^R70K^* was expressed from a low-copy plasmid. B) Either *IQG1* or *iqg1^A654V^* was expressed from a low copy plasmid in an *iqg1Δ* strain expressing Mup1-pHl. Mup1-pHl images were acquired 45 min after addition of 20 μg/ml methionine. Scale bar, 2μm. C) Schematics of *Saccharomyces cerevisiae* (Yeast) Kre33 and Human homolog Nat10 protein domains showing an alignment surrounding the mutation (black arrow) using Pfam and Sequence Manipulation Suite (Stothard 2000; Finn *et al.* 2016) D) Schematics of *Saccharomyces cerevisiae* (Yeast) Iqg1 and Human homolog IQGAP3 protein domains showing an alignment surrounding the mutation (black arrow) using Pfam and Sequence Manipulation Suite. Amino acids included within IQ motifs are indicated with black bars; below for Human IQGAP3 and above for Yeast Iqg1. E) Either *KRE33* or *kre33^R70K^* was expressed from a low-copy plasmid in *kre33Δ* cells expressing Abp1-RFP. Arrow indicates aberrant actin patches. F) In an *iqg1Δ* strain expressing Abp1-RFP, either *IQG1* or *iqg1^A654V^* was expressed from a low copy plasmid. Arrows indicate aberrant actin patches. G) Lifetimes of Ede1-GFP, Pan1-GFP and Abp1-RFP calculated from movies of 120 frames acquired at intervals of 3 s (Syp1- GFP) or 2 s (Pan1-GFP, Abp1-RFP) (N > 50 patches/strain). The central lines of the box plot correspond to the median and the whiskers denote the min and max lifetimes. * *P* < 0.05 using an F test.

Using whole genome sequencing of mutant strains, we identified point mutations in *KRE33* and *IQG1*, two essential and conserved genes encoding proteins that have never been previously implicated in endocytosis. *KRE33* encodes an acetyl transferase involved in the small ribosomal subunit folding complex that acetylates 18S rRNA, as well as tRNAs (Li *et al.* 2009; Ito *et al.* 2014). The mammalian homolog of Kre33, NAT10, acetylates proteins as well as RNAs; however, acetylation of proteins by yeast Kre33 has not yet been demonstrated (Shen *et al.* 2009; Larrieu *et al.* 2014; Ito *et al.* 2014; Sharma *et al.* 2015). *IQG1* is the yeast homolog of IQGAP, which is a downstream effector of Rho GTPases that directly binds to F-actin and scaffolds actin regulatory proteins (Watanabe *et al.* 2015). Yeast Iqg1 localizes to the bud neck and plays important roles in polarized secretion and cytokinesis (Epp and Chant 1997; Lippincott and Li 1998; Osman and Cerione 1998). The mutations we identified in *KRE33* and *IQG1* each change a single amino acid that produces an endocytic defect; arginine 70 was mutated to a lysine in Kre33 (*kre33^R70K^*) and alanine 654 was mutated to a valine in Iqg1 (*iqg1^A654V^*). We engineered these point mutations individually into the parental strain, which resulted in an endocytic defect in the Mup1-pHl endocytosis assay (Figure 7, A and B). Both Kre33 and Iqg1 show similar domain structure compared to their human homologs, NAT10 and IQGAP3, respectively (Figure 7C,D). In both cases, the amino acid that was altered in the mutant is not only conserved, but also positioned within a relatively highly conserved region of the protein.

Aberrant actin phenotypes are observed in many cells defective in CME. For example, actin-uncoupling phenotypes are seen in *sla2Δ* or *ent1*/*2Δ* cells with short actin comet tails, uncontrolled Arp2/3 actin plumes are observed in *sla1Δ/bbc1Δ* or *end3Δ/bbc1Δ* cells, and an intermediate actin flare phenotype is seen in Pan1-depleted cells (Kaksonen *et al.* 2003; 2005; Skruzny *et al.* 2012; Bradford *et al.* 2015). Similar to other CME defective strains, *kre33^R70K^* and *iqg1^A654V^*cells show aberrant actin filament formation at a subset of endocytic sites using Abp1-RFP localization (Figure 7E,F). The average lifetimes of Ede1-GFP, Pan1-GFP, and Abp1-RFP at endocytic sites were not significantly different in the mutant *kre33^R70K^* and *iqg1^A654V^* strains (Figure 7G). Interestingly, however, the variance in the distribution of the endocytic patch lifetimes of Pan1-GFP and Abp1-RFP exhibited a statistically significant increase in variability for both mutant strains as compared to their WT counterparts. These data further support Iqg1 and Kre33 playing a role in regulation of the endocytic machinery.

## Discussion

CME is a well-studied process in both yeast and mammalian cells. Previous studies have identified roles for many proteins in CME; however, there are likely additional, as-yet unidentified factors that contribute to endocytic events. Using a combination of EMS mutagenesis and flow cytometry, we isolated 256 mutants with defects representing steps throughout the entire endocytic and endosomal trafficking process. The direct approach of using flow cytometry to assay endocytic function and to quantitatively monitor internalization of a specific endocytic cargo protein (Mup1) facilitated our identification and analysis of a large number of mutants. Utilizing EMS to introduce random point mutations allowed us to generate an unbiased collection of mutant strains that contained truncation and missense alleles, which could provide clues to functional amino acid(s) and regions of proteins that are important for CME that would otherwise go unnoticed when using the yeast knockout collection. Temperature sensitivity has been used as a secondary phenotype linked to mutant defects in previous genetic screens (Raths 1993; Wendland *et al.* 1996), but only 29 mutant strains were temperature sensitive. We tested the seven temperature sensitive mutants within the plasma membrane class; one of these contained the *kre33^R70K^* mutant, but the temperature sensitivity was unlinked to the *KRE33* locus. The remaining six were not complemented by any of our candidate endocytosis genes, and the causative mutation remains uncharacterized in these cells. Since our screen directly assessed cargo internalization without relying on a secondary phenotype, it is likely that many mutants isolated in this study represent alleles that were not isolated in previous genetic screens.

This study focused on characterizing the recessive, PM-localized class of endocytic mutants. This class was further organized into four subclasses based on the ability to internalize multiple cargos: Mup1, Snc1, Ste3, and Fur4. We expected to find a subclass of mutants with endocytic defects specific to Mup1 internalization, since Mup1-pHl formed the basis for mutant selection in our screen. However, none of the factors previously implicated in Mup1 internalization, specifically (Ldb19, Rsp5, Npr1), were mutated in strains from the Mup1-selective class of endocytic mutants. Expression of additional copies of *LDB19* suppressed three Mup1-selective mutant strains and four permease-selective mutant strains, even though sequencing demonstrated that the endogenous *LDB19* gene was not mutated in these strains. Ldb19 is an arrestin-like protein, and there are thirteen α- related arrestins in yeast that recruit the Rsp5 ubiquitin ligase, which promotes the internalization of specific cargo proteins (Lin *et al.* 2008). One possible explanation for this is that extra copies of *LDB19* are able to bypass the need for an unidentified mutant factor by facilitating the marking of Mup1 for internalization; such factors may include other arrestin-like proteins that play partially redundant roles in internalization of some endocytic cargos. Further investigation is needed to determine how Ldb19 is able to suppress several distinct mutations to promote internalization of Mup1 and to identify the causative allele(s) in these mutant strains.

Importantly, we identified truncations in the known endocytic genes *SLA2*, *SLA1*, and *EDE1*, which validated our screening method. Those truncations can provide further mechanistic insights into how these proteins facilitate the endocytic process. Ede1 is one of the earliest coat proteins to localize at nascent endocytic sites, and the *ede1^1-318^* truncation results in a shorter lifetime for both Pan1-GFP and Abp1-RFP at cortical actin patches. This phenotype resembles defects in actin patch protein dynamics seen in *ede1*∆ cells (Kaksonen *et al.* 2005; Newpher and Lemmon 2006). A recent study showed that seven of the earliest-arriving endocytic proteins are expendable during endocytosis in yeast cells, including Ede1, because lipid uptake is normal (Brach *et al.* 2014). However, these early factors are required for nutrient-induced endocytosis of the cargo protein Dip5. In our study, we found that Ede1^1-318^ produces defects in nutrient-regulated endocytosis of Mup1 and in constitutive endocytosis of Snc1 and Ste3, which suggests that Ede1 is not dispensable in endocytosis of specific cargos as was previously thought.

Arp3 is an essential gene required for branched actin network formation through the actin nucleating activity of the Arp2/3 complex. Arp2/3-mediated actin assembly is a major driving force in membrane deformation at CME sites in yeast, leading to invagination and scission; these branched actin structures are required to overcome the high turgor pressure at the plasma membrane within yeast cells (Aghamohammadzadeh and Ayscough 2009). We found that Arp2/Arp3^R346H^ has a severe defect in nucleating branched actin filaments *in vitro*. Arginine (WT) and histidine (mutant) are both polar, positively-charged amino acids. We propose that the histidine residue in the mutant arp3 protein weakens its interaction with the actin monomer, resulting in the nucleation defect. Arp3^R346H^ likely supports sufficient nucleation activity for cell viability, as seen by reduced, but not abolished, localization of Arc15 and Abp1 to cortical patches. The endocytic machinery localizes normally but is arrested at unproductive sites, presumably because Arp2/Arp3^R346H^ complex is a poor nucleator of actin filaments. The reduced nucleation of these actin filaments is likely insufficient for efficient completion of endocytic events. Similar mutants in Arp2 have been isolated, such as *arp2-1*, which alters one residue at the barbed end of Arp2 and causes defects in actin nucleation *in vitro* and endocytosis *in vivo* (Moreau *et al.* 1997; D’Agostino and Goode 2005; Boettner *et al.* 2009). Thus, both *arp2-1* and *arp3^R346H^* mutations reside at the barbed end of the proteins, and each mutant gives rise to defects in endocytosis. However, no shared genetic interactions have been identified to date (for example, *las17*Δ or *myo3*Δ *myo5*Δ) between the *arp2-1* and *arp3^R346H^* mutants, and the endocytic protein lifetime phenotypes are distinct (Moreau *et al.* 1997; D’Agostino and Goode 2005; Boettner *et al.* 2009).

Performing an EMS mutagenesis screen allowed us to identify missense mutations in the essential genes *KRE33* and *IQG1* that resulted in previously unreported endocytic defects. These genes are among the ∼1,500 essential genes excluded from the yeast knockout collection and thus, have been overlooked by studies using such collections. Moreover, missense mutations in these large proteins point to functional domains that may contribute to CME. Both genes are conserved from yeast to humans, and the mutations that cause defects in endocytosis reside within highly-conserved regions of the proteins. Kre33 has been characterized as a protein involved in the small ribosomal subunit folding complex, and it was also identified in a genetic screen for mutants resistant to the K1 killer toxin (Pagé *et al.* 2003; Li *et al.* 2009); interestingly, changes in endocytosis could affect the ability of the toxin to kill. *KRE33* was recently proposed to be renamed *RRA1* because it encodes an acetyl transferase that acetylates 18S rRNA, as well as tRNAs. Its homolog, NAT10, performs similar functions in mammalian cells (Ito *et al.* 2014) where it also acetylates histone and microtubule proteins (Shen *et al.* 2009; Larrieu *et al.* 2014; Sharma *et al.* 2015). In a recent paper, a tubulin mutant that was incapable of binding Bik1, the yeast ortholog of mammalian CLIP-170, was unable to traffic Snc1 efficiently (Boscheron *et al.* 2016). Moreover, yeast cells expressing mutant tubulin displayed an actin flare phenotype reminiscent of previously described endocytic mutants (Kaksonen *et al.* 2003; 2005; Skruzny *et al.* 2012; Bradford *et al.* 2015; Boscheron *et al.* 2016); similar aberrant actin phenotypes were also observed in *kre33^R70K^* and *iqg1^A654V^* cells. Thus, cross-talk between the microtubules and the actin cytoskeleton may play a previously unrecognized role in yeast membrane trafficking. The increase in variability for the lifetimes of Pan1 and Abp1 in the *kre33^R70K^* and *iqg1^A654V^* mutant strains could reveal a loss of regulation that leads to poor coordination at endocytic sites. A loss of synchronization would likely lead to a more random endocytic process. In yeast cells, it has been shown that CME is more consistent in polarized cells as compared to non-polarized cells; further, the polarized endocytic sites are the most rapidly maturing early coat patches (Layton *et al.* 2011; Jose *et al.* 2013). Another study demonstrated heterogeneity in the dynamics of proteins at endocytic sites, and the authors speculated a checkpoint governed by cargo could be a mechanism to control the rate of endocytosis (Loerke *et al.* 2009). Iqg1 and Kre33 mutants causing the increase in variation of endocytic protein lifetimes at the plasma membrane may be lacking the full regulation needed for the highly precise coordination that is characteristic of CME.

kre33^R70K^ may affect endocytosis due to a general reduction in translation or by reducing the ability to fine-tune translation because the ribosome is unable to assemble correctly. However, neither explanation seems likely because the mutant does not have observable growth defects (K. Wrasman, and B. Wendland, unpublished data). Perhaps Kre33 directly acetylates endocytic proteins to regulate their functions; further studies are needed to determine if Kre33 can acetylate proteins similar to its mammalian homolog, NAT10 (Shen *et al.* 2009). Alternatively, Kre33 may acetylate a putative RNA with an unrecognized role in the endocytic process. Future studies of the mutant protein and characterization of the endocytic defect in *kre33^R70K^* cells will provide better insights into the specific endocytic functions of Kre33.

*IQG1* is homologous to the mammalian IQGAP proteins, which are Rho GTPase effectors that regulate actin cytoskeleton functions. These proteins have an N-terminal Calponin homology domain (CHD) that binds and bundles F-actin, calmodulin-binding IQ motifs, and a Ras-GAP domain (RGD) at the C-terminus that binds directly to actin filament barbed ends, formins, APC, and CLIP-170. In mammalian cells, IQGAP1 acts both upstream and downstream of mTORC1 to regulate cellular homeostasis and to connect cell growth and division (Tekletsadik *et al.* 2012). Similarly, Iqg1 may contribute to endocytosis in yeast cells by regulating signaling through the environmental sensors Tor1 and Tor2 (deHart *et al.* 2003). Alternatively, the A654V missense mutation is located in the IQ repeat region of Iqg1, and the IQ repeats are required for Myo1 localization to the contractile ring during cytokinesis in yeast (Boyne *et al.* 2000). Myo1 is also important for secretory vesicle targeting during cytokinesis; therefore, a mutation that causes a defect in Myo1 localization may affect exocytosis and consequently, result in a compensatory endocytic defect (Osman *et al.* 2002). Determining how the mutant form of Iqg1 functions in endocytosis will require future study.

This phenotype-based genetic screen yielded new loss-of-function truncations in well-established CME machinery components and additionally revealed novel endocytic roles for essential genes that have not been previously implicated in CME. Further study of these genes, as well as characterizing other mutants in our collection, will surely shed new light on endocytic mechanism and regulation.

## References

[bib1] Aghamohammadzadeh S. Ayscough K. R., 2009 Differential requirements for actin during yeast and mammalian endocytosis. Nature Publishing Group 11: 1039–1042. 10.1038/ncb1918 PMC2875176 19597484

[bib2] Aguilar R. C. Longhi S. A. Shaw J. D. Yeh L.-Y. Kim S., 2006 Epsin N-terminal homology domains perform an essential function regulating Cdc42 through binding Cdc42 GTPase-activating proteins. Proc. Natl. Acad. Sci. USA 103(11): 4116–4121. 10.1073/pnas.0510513103 16537494 PMC1449656

[bib3] Ayscough K. R. Stryker J. Pokala N. Sanders M. Crews P., 1997 High rates of actin filament turnover in budding yeast and roles for actin in establishment and maintenance of cell polarity revealed using the actin inhibitor latrunculin-A. J. Cell Biol. 137(2): 399–416. 10.1083/jcb.137.2.399 9128251 PMC2139767

[bib4] Barker S. L. Lee L. Pierce B. D. Maldonado-Baez L. Drubin D. G., 2007 Interaction of the endocytic scaffold protein Pan1 with the type I myosins contributes to the late stages of endocytosis. Mol. Biol. Cell 18(8): 2893–2903. 10.1091/mbc.E07-05-0436 17522383 PMC1949359

[bib5] Bitsikas V. Corrêa I. R. Nichols B. J., 2014 Clathrin-independent pathways do not contribute significantly to endocytic flux. eLife 3: e03970 10.7554/eLife.03970 25232658 PMC4185422

[bib6] Blondel M.-O. Morvan J. Dupré S. Urban-Grimal D. Haguenauer-Tsapis R., 2004 Direct sorting of the yeast uracil permease to the endosomal system is controlled by uracil binding and Rsp5p-dependent ubiquitylation. Mol. Biol. Cell 15(2): 883–895. 10.1091/mbc.E03-04-0202 14657252 PMC329401

[bib7] Boettner D. R. Agostino J. L. D. Torres O. T. Daugherty-Clarke K. Uygur A., 2009 The F-BAR Protein Syp1 Negatively Regulates WASp-Arp2/3 Complex Activity during Endocytic Patch Formation. Curr. Biol. 19(23): 1979–1987. 10.1016/j.cub.2009.10.062 19962315 PMC2828323

[bib8] Boettner D. R. Chi R. J. Lemmon S. K., 2012 Lessons from yeast for clathrin-mediated endocytosis. Nature Publishing Group 14: 2–10. 10.1038/ncb2403 PMC5590828 22193158

[bib9] Bokman S. H. Ward W. W., 1981 Renaturation of Aequorea gree-fluorescent protein. Biochem. Biophys. Res. Commun. 101(4): 1372–1380. 10.1016/0006-291X(81)91599-0 7306136

[bib10] Boscheron C. Caudron F. Loeillet S. Peloso C. Mugnier M., 2016 A role for the yeast CLIP170 ortholog, the plus-end-tracking protein Bik1, and the Rho1 GTPase in Snc1 trafficking. J. Cell Sci. 129(17): 3332–3341. 10.1242/jcs.190330 27466378 PMC5047699

[bib11] Boyne J. R. Yosuf H. M. Bieganowski P. Brenner C. Price C., 2000 Yeast myosin light chain, Mlc1p, interacts with both IQGAP and class II myosin to effect cytokinesis. J. Cell Sci. 113(Pt 24): 4533–4543. 11082046 10.1242/jcs.113.24.4533

[bib12] Brach T. Godlee C. Moeller-Hansen I. Boeke D. Kaksonen M., 2014 The Initiation of Clathrin-Mediated Endocytosis Is Mechanistically Highly Flexible. Curr. Biol. 24(5): 548–554. 10.1016/j.cub.2014.01.048 24530066

[bib13] Bradford M. K. Whitworth K. Wendland B., 2015 Pan1 regulates transitions between stages of clathrin-mediated endocytosis. Mol. Biol. Cell 26(7): 1371–1385. 10.1091/mbc.E14-11-1510 25631817 PMC4454182

[bib14] Burston H. E. Maldonado-Baez L. Davey M. Montpetit B. Schluter C., 2009 Regulators of yeast endocytosis identified by systematic quantitative analysis. J. Cell Biol. 185(6): 1097–1110. 10.1083/jcb.200811116 19506040 PMC2711619

[bib15] Carroll S. Y. Stimpson H. E. M. Weinberg J. Toret C. P. Sun Y., 2012 Analysis of yeast endocytic site formation and maturation through a regulatory transition point. Mol. Biol. Cell 23(4): 657–668. 10.1091/mbc.E11-02-0108 22190733 PMC3279393

[bib16] Chen L. Davis N. G., 2000 Recycling of the yeast a-factor receptor. J. Cell Biol. 151(3): 731–738. 10.1083/jcb.151.3.731 11062272 PMC2185590

[bib17] Chi R. J. Torres O. T. Segarra V. A. Lansley T. Chang J. S., 2012 Role of Scd5, a protein phosphatase-1 targeting protein, in phosphoregulation of Sla1 during endocytosis. J. Cell Sci. 125(20): 4728–4739. 10.1242/jcs.098871 22825870 PMC3517093

[bib18] D’Agostino J. L. Goode B. L., 2005 Dissection of Arp2/3 complex actin nucleation mechanism and distinct roles for its nucleation-promoting factors in Saccharomyces cerevisiae. Genetics 171(1): 35–47. 10.1534/genetics.105.040634 16183906 PMC1456526

[bib19] Danecek P. Auton A. Abecasis G. Albers C. A. Banks E., 2011 The variant call format and VCFtools. Bioinformatics 27(15): 2156–2158. 10.1093/bioinformatics/btr330 21653522 PMC3137218

[bib20] De Craene J.-O. Soetens O. André B., 2001 The Npr1 Kinase Controls Biosynthetic and Endocytic Sorting of the Yeast Gap1 Permease. J. Biol. Chem. 276(47): 43939–43948. 10.1074/jbc.M102944200 11500493

[bib21] deHart A. K. A. Schnell J. D. Allen D. A. Tsai J.-Y. Hicke L., 2003 Receptor internalization in yeast requires the Tor2-Rho1 signaling pathway. Mol. Biol. Cell 14(11): 4676–4684. 10.1091/mbc.E03-05-0323 14593073 PMC266782

[bib22] DiPrima S. Haarer B. Viggiano S. Pons C. Myers C. L., 2014 Linking genetics to structural biology: complex heterozygosity screening with actin alanine scan alleles identifies functionally related surfaces on yeast actin. G3 (Bethesda) 4(8): 1491–1501. 10.1534/g3.114.012054 24938290 PMC4132179

[bib23] Epp J. A. Chant J., 1997 An IQGAP-related protein controls actin-ring formation and cytokinesis in yeast. Curr. Biol. 7(12): 921–929. 10.1016/S0960-9822(06)00411-8 9382845

[bib24] Farrell K. B. Grossman C. Di Pietro S. M., 2015 New Regulators of Clathrin-Mediated Endocytosis Identified in *Saccharomyces cerevisiae* by Systematic Quantitative Fluorescence Microscopy. Genetics 201(3): 1061–1070. 10.1534/genetics.115.180729 26362318 PMC4649635

[bib26] Finn R. D. Coggill P. Eberhardt R. Y. Eddy S. R. Mistry J., 2016 The Pfam protein families database: towards a more sustainable future. Nucleic Acids Res. 44(D1): D279–D285. 10.1093/nar/gkv1344 26673716 PMC4702930

[bib27] Gagny B. Wiederkehr A. Dumoulin P. Winsor B. Riezman H., 2000 A novel EH domain protein of Saccharomyces cerevisiae, Ede1p, involved in endocytosis. J. Cell Sci. 113(Pt 18): 3309–3319. 10954428 10.1242/jcs.113.18.3309

[bib28] Gardiner F. C. Costa R. Ayscough K. R., 2007 Nucleocytoplasmic trafficking is required for functioning of the adaptor protein Sla1p in endocytosis. Traffic 8(4): 347–358. 10.1111/j.1600-0854.2007.00534.x 17286805 PMC1989034

[bib29] Goode B. L. Eskin J. A. Wendland B., 2015 Actin and Endocytosis in Budding Yeast. Genetics 199(2): 315–358. 10.1534/genetics.112.145540 25657349 PMC4317646

[bib30] Gurunathan S. Chapman-Shimshoni D. Trajkovic S. Gerst J. E., 2000 Yeast exocytic v-SNAREs confer endocytosis. Mol. Biol. Cell 11(10): 3629–3643. 10.1091/mbc.11.10.3629 11029060 PMC15020

[bib31] Hicke L. Riezman H., 1996 Ubiquitination of a yeast plasma membrane receptor signals its ligand-stimulated endocytosis. Cell 84(2): 277–287. 10.1016/S0092-8674(00)80982-4 8565073

[bib32] Holtzman D. A. Yang S. Drubin D. G., 1993 Synthetic-lethal interactions identify two novel genes, SLA1 and SLA2, that control membrane cytoskeleton assembly in Saccharomyces cerevisiae. J. Cell Biol. 122(3): 635–644. 10.1083/jcb.122.3.635 8335689 PMC2119656

[bib33] Isnard A. Thomas D. Surdin-Kerjan Y., 1996 The Study of Methionine Uptake inSaccharomyces cerevisiaeReveals a New Family of Amino Acid Permeases. J. Mol. Biol. 262(4): 473–484. 10.1006/jmbi.1996.0529 8893857

[bib34] Ito S. Akamatsu Y. Noma A. Kimura S. Miyauchi K., 2014 A Single Acetylation of 18 S rRNA Is Essential for Biogenesis of the Small Ribosomal Subunit in Saccharomyces cerevisiae. J. Biol. Chem. 289(38): 26201–26212. 10.1074/jbc.M114.593996 25086048 PMC4176211

[bib35] Jones C. B. Ott E. M. Keener J. M. Curtiss M. Sandrin V., 2012 Regulation of membrane protein degradation by starvation-response pathways. Traffic 13: 468–482. 10.1111/j.1600-0854.2011.01314.x 22118530 PMC3276697

[bib36] Jose M. Tollis S. Nair D. Sibarita J. B. McCusker D., 2013 Robust polarity establishment occurs via an endocytosis-based cortical corralling mechanism. J. Cell Biol. 200(4): 407–418. 10.1083/jcb.201206081 23401000 PMC3575534

[bib37] Kaksonen M. Sun Y. Drubin D. G., 2003 A pathway for association of receptors, adaptors, and actin during endocytic internalization. Cell 115(4): 475–487. 10.1016/S0092-8674(03)00883-3 14622601

[bib38] Kaksonen M. Toret C. P. Drubin D. G., 2005 A modular design for the clathrin- and actin-mediated endocytosis machinery. Cell 123(2): 305–320. 10.1016/j.cell.2005.09.024 16239147

[bib39] Kaksonen M. Toret C. P. Drubin D. G., 2006 Harnessing actin dynamics for clathrin-mediated endocytosis. Nat. Rev. Mol. Cell Biol. 7(6): 404–414. 10.1038/nrm1940 16723976

[bib40] Kalthoff C. Alves J. Urbanke C. Knorr R. Ungewickell E. J., 2002 Unusual structural organization of the endocytic proteins AP180 and epsin 1. J. Biol. Chem. 277(10): 8209–8216. 10.1074/jbc.M111587200 11756460

[bib41] Kaouass M. Gamache I. Ramotar D. Audette M. Poulin R., 1998 The spermidine transport system is regulated by ligand inactivation, endocytosis, and by the Npr1p Ser/Thr protein kinase in Saccharomyces cerevisiae. J. Biol. Chem. 273(4): 2109–2117. 10.1074/jbc.273.4.2109 9442051

[bib42] Keener J. M. Babst M., 2013 Quality Control and Substrate-Dependent Downregulation of the Nutrient Transporter Fur4. Traffic 14(4): 412–427. 10.1111/tra.12039 23305501 PMC3594327

[bib43] Kuhn J. R. Pollard T. D., 2005 Real-time measurements of actin filament polymerization by total internal reflection fluorescence microscopy. Biophys. J. 88(2): 1387–1402. 10.1529/biophysj.104.047399 15556992 PMC1305141

[bib44] Kumari S. Mg S. Mayor S., 2010 Endocytosis unplugged: multiple ways to enter the cell. Cell Res. 20(3): 256–275. 10.1038/cr.2010.19 20125123 PMC7091825

[bib45] Larrieu D. Britton S. Demir M. Rodriguez R. Jackson S. P., 2014 Chemical inhibition of NAT10 corrects defects of laminopathic cells. Science 344(6183): 527–532. 10.1126/science.1252651 24786082 PMC4246063

[bib46] Layton A. T. Savage N. S. Howell A. S. Carroll S. Y. Drubin D. G., 2011 Modeling vesicle traffic reveals unexpected consequences for Cdc42p-mediated polarity establishment. Curr. Biol. 21(3): 184–194. 10.1016/j.cub.2011.01.012 21277209 PMC3052744

[bib47] Lee W. R. Beranek D. T. Byrne B. J. Tucker A. B., 1990 Comparison of dose-response relationships for ethyl methanesulfonate and 1-ethyl-1-nitrosourea in Drosophila melanogaster spermatozoa. Mutat. Res. 231(1): 31–45. 10.1016/0027-5107(90)90174-3 2114533

[bib48] Lewis M. J. Nichols B. J. Prescianotto-Baschong C. Riezman H. Pelham H. R., 2000 Specific retrieval of the exocytic SNARE Snc1p from early yeast endosomes. Mol. Biol. Cell 11(1): 23–38. 10.1091/mbc.11.1.23 10637288 PMC14754

[bib49] Li Z. Lee I. Moradi E. Hung N. J. Johnson A. W., 2009 Rational extension of the ribosome biogenesis pathway using network-guided genetics. PLoS Biol. 7: e1000213 10.1371/journal.pbio.1000213 19806183 PMC2749941

[bib50] Lin C. H. MacGurn J. A. Chu T. Stefan C. J. Emr S. D., 2008 Arrestin-related ubiquitin-ligase adaptors regulate endocytosis and protein turnover at the cell surface. Cell 135(4): 714–725. 10.1016/j.cell.2008.09.025 18976803

[bib51] Lippincott J. Li R., 1998 Sequential assembly of myosin II, an IQGAP-like protein, and filamentous actin to a ring structure involved in budding yeast cytokinesis. J. Cell Biol. 140(2): 355–366. 10.1083/jcb.140.2.355 9442111 PMC2132585

[bib52] Loerke D. Mettlen M. Yarar D. Jaqaman K. Jaqaman H., 2009 Cargo and dynamin regulate clathrin-coated pit maturation. PLoS Biol. 7(3): e57 10.1371/journal.pbio.1000057 19296720 PMC2656549

[bib53] Longtine M. S. McKenzie A. Demarini D. J. Shah N. G. Wach A., 1998 Additional modules for versatile and economical PCR-based gene deletion and modification in Saccharomyces cerevisiae. Yeast 14(10): 953–961. 10.1002/(SICI)1097-0061(199807)14:10<953::AID-YEA293>3.0.CO;2-U 9717241

[bib54] Lu R. Drubin D. G., 2017 Selection and stabilization of endocytic sites by Ede1, a yeast functional homologue of human Eps15. Mol. Biol. Cell 28: 567–575. 10.1091/mbc.E16-06-0391 28057762 PMC5328616

[bib55] MacGurn J. A. Hsu P.-C. Smolka M. B. Emr S. D., 2011 TORC1 Regulates Endocytosis via Npr1-Mediated Phosphoinhibition of a Ubiquitin Ligase Adaptor. Cell 147(5): 1104–1117. 10.1016/j.cell.2011.09.054 22118465

[bib56] Machesky L. M., 1998 Cytokinesis: IQGAPs find a function. Curr. Biol. 8(6): R202–R205. 10.1016/S0960-9822(98)70125-3 9512410

[bib57] Maldonado-Baez L. Dores M. R. Perkins E. M. Drivas T. G. Hicke L., 2008 Interaction between Epsin/Yap180 adaptors and the scaffolds Ede1/Pan1 is required for endocytosis. Mol. Biol. Cell 19(7): 2936–2948. 10.1091/mbc.E07-10-1019 18448668 PMC2441688

[bib58] McLaren W. Gil L. Hunt S. E. Riat H. S. Ritchie G. R. S., 2016 The Ensembl Variant Effect Predictor. Genome Biol. 17(1): 122 10.1186/s13059-016-0974-4 27268795 PMC4893825

[bib59] Michelot A. Costanzo M. Sarkeshik A. Boone C. Yates J. R., 2010 Reconstitution and protein composition analysis of endocytic actin patches. Curr. Biol. 20(21): 1890–1899. 10.1016/j.cub.2010.10.016 21035341 PMC2998891

[bib60] Miesenböck G. De Angelis D. A. Rothman J. E., 1998 Visualizing secretion and synaptic transmission with pH-sensitive green fluorescent proteins. Nature 394(6689): 192–195. 10.1038/28190 9671304

[bib61] Moreau V. Galan J. M. Devilliers G. Haguenauer-Tsapis R. Winsor B., 1997 The yeast actin-related protein Arp2p is required for the internalization step of endocytosis. Mol. Biol. Cell 8(7): 1361–1375. 10.1091/mbc.8.7.1361 9243513 PMC276158

[bib62] Newpher T. M. Lemmon S. K., 2006 Clathrin is important for normal actin dynamics and progression of Sla2p-containing patches during endocytosis in yeast. Traffic 7(5): 574–588. 10.1111/j.1600-0854.2006.00410.x 16643280 PMC2975023

[bib63] Newpher T. M. Smith R. P. Lemmon V. Lemmon S. K., 2005 In vivo dynamics of clathrin and its adaptor-dependent recruitment to the actin-based endocytic machinery in yeast. Dev. Cell 9(1): 87–98. 10.1016/j.devcel.2005.04.014 15992543

[bib64] Nikko E. Pelham H. R. B., 2009 Arrestin-mediated endocytosis of yeast plasma membrane transporters. Traffic 10(12): 1856–1867. 10.1111/j.1600-0854.2009.00990.x 19912579 PMC2810449

[bib65] Osman M. A. Cerione R. A., 1998 Iqg1p, a yeast homologue of the mammalian IQGAPs, mediates cdc42p effects on the actin cytoskeleton. J. Cell Biol. 142(2): 443–455. 10.1083/jcb.142.2.443 9679143 PMC2133066

[bib66] Osman M. A. Konopka J. B. Cerione R. A., 2002 Iqg1p links spatial and secretion landmarks to polarity and cytokinesis. J. Cell Biol. 159(4): 601–611. 10.1083/jcb.200205084 12446742 PMC2173104

[bib67] Pagé N. Gérard-Vincent M. Ménard P. Beaulieu M. Azuma M., 2003 A Saccharomyces cerevisiae genome-wide mutant screen for altered sensitivity to K1 killer toxin. Genetics 163: 875–894. 12663529 10.1093/genetics/163.3.875 PMC1462477

[bib68] Pettersen E. F. Goddard T. D. Huang C. C. Couch G. S. Greenblatt D. M., 2004 UCSF Chimera–a visualization system for exploratory research and analysis. J. Comput. Chem. 25(13): 1605–1612. 10.1002/jcc.20084 15264254

[bib69] Pollard T. D. Cooper J. A., 1984 Quantitative analysis of the effect of Acanthamoeba profilin on actin filament nucleation and elongation. Biochemistry 23(26): 6631–6641. 10.1021/bi00321a054 6543322

[bib70] Prosser D. C. Wendland B., 2016 DePFth Perception in Clathrin-Mediated Endocytosis. Dev. Cell 37(5): 387–388. 10.1016/j.devcel.2016.05.017 27270034

[bib71] Prosser D. C. Drivas T. G. Maldonado-Baez L. Wendland B., 2011 Existence of a novel clathrin-independent endocytic pathway in yeast that depends on Rho1 and formin. J. Cell Biol. 195(4): 657–671. 10.1083/jcb.201104045 22065638 PMC3257529

[bib72] Prosser D. C. Pannunzio A. E. Brodsky J. L. Thorner J. Wendland B., 2015 -Arrestins participate in cargo selection for both clathrin-independent and clathrin-mediated endocytosis. J. Cell Sci. 128(22): 4220–4234. 10.1242/jcs.175372 26459639 PMC4712785

[bib73] Prosser D. C. Whitworth K. Wendland B., 2010 Quantitative Analysis of Endocytosis with Cytoplasmic pHluorin Chimeras. Traffic 11(9): 1141–1150. 10.1111/j.1600-0854.2010.01088.x 20626707 PMC2919640

[bib74] Prosser D. C. Wrasman K. Woodard T. K. O’Donnell A. F. Wendland B., 2016 Applications of pHluorin for Quantitative, Kinetic and High-throughput Analysis of Endocytosis in Budding Yeast. J. Vis. Exp. (116), e54587, doi:10:3791/54587 10.3791/54587 PMC5092240 27805610

[bib75] Raths S., 1993 end3 and end4: two mutants defective in receptor-mediated and fluid- phase endocytosis in Saccharomyces cerevisiae. J. Cell Biol. 120(1): 55–65. 10.1083/jcb.120.1.55 8380177 PMC2119492

[bib76] Raymond C. K. Howald-Stevenson I. Vater C. A. Stevens T. H., 1992 Morphological classification of the yeast vacuolar protein sorting mutants: evidence for a prevacuolar compartment in class E vps mutants. Mol. Biol. Cell 3(12): 1389–1402. 10.1091/mbc.3.12.1389 1493335 PMC275707

[bib77] Reider A. Barker S. L. Mishra S. K. Im Y. J. Maldonado-Báez L., 2009 Syp1 is a conserved endocytic adaptor that contains domains involved in cargo selection and membrane tubulation. EMBO J. 28(20): 3103–3116. 10.1038/emboj.2009.248 19713939 PMC2771086

[bib78] Roth A. F. Davis N. G., 1996 Ubiquitination of the yeast a-factor receptor. J. Cell Biol. 134(3): 661–674. 10.1083/jcb.134.3.661 8707846 PMC2120937

[bib79] Sankaranarayanan S. De Angelis D. Rothman J. E. Ryan T. A., 2000 The use of pHluorins for optical measurements of presynaptic activity. Biophys. J. 79(4): 2199–2208. 10.1016/S0006-3495(00)76468-X 11023924 PMC1301110

[bib80] Sharma S. Langhendries J. L. Watzinger P. Kotter P. Entian K. D., 2015 Yeast Kre33 and human NAT10 are conserved 18S rRNA cytosine acetyltransferases that modify tRNAs assisted by the adaptor Tan1/THUMPD1. Nucleic Acids Res. 43(4): 2242–2258. 10.1093/nar/gkv075 25653167 PMC4344512

[bib81] Shen Q. Zheng X. McNutt M. A. Guang L. Sun Y., 2009 NAT10, a nucleolar protein, localizes to the midbody and regulates cytokinesis and acetylation of microtubules. Exp. Cell Res. 315(10): 1653–1667. 10.1016/j.yexcr.2009.03.007 19303003

[bib82] Shih S. C. Katzmann D. J. Schnell J. D. Sutanto M. Emr S. D., 2002 Epsins and Vps27p/Hrs contain ubiquitin-binding domains that function in receptor endocytosis. Nat. Cell Biol. 4(5): 389–393. 10.1038/ncb790 11988742

[bib83] Skruzny M. Brach T. Ciuffa R. Rybina S. Wachsmuth M., 2012 Molecular basis for coupling the plasma membrane to the actin cytoskeleton during clathrin-mediated endocytosis. Proc. Natl. Acad. Sci. USA 109(38): E2533–E2542. 10.1073/pnas.1207011109 22927393 PMC3458359

[bib84] Smith B. A. Daugherty-Clarke K. Goode B. L. Gelles J., 2013 Pathway of actin filament branch formation by Arp2/3 complex revealed by single-molecule imaging. Proc. Natl. Acad. Sci. USA 110(4): 1285–1290. 10.1073/pnas.1211164110 23292935 PMC3557048

[bib85] Spudich J. A. Watt S., 1971 The regulation of rabbit skeletal muscle contraction. I. Biochemical studies of the interaction of the tropomyosin-troponin complex with actin and the proteolytic fragments of myosin. J. Biol. Chem. 246: 4866–4871. 4254541

[bib86] Stimpson H. E. M. Toret C. P. Cheng A. T. Pauly B. S. Drubin D. G., 2009 Early-arriving Syp1p and Ede1p function in endocytic site placement and formation in budding yeast. Mol. Biol. Cell 20(22): 4640–4651. 10.1091/mbc.E09-05-0429 19776351 PMC2777095

[bib87] Stothard P., 2000 The sequence manipulation suite: JavaScript programs for analyzing and formatting protein and DNA sequences. Biotechniques 28: 1102–1104. 10868275 10.2144/00286ir01

[bib88] Sun Y. Carroll S. Kaksonen M. Toshima J. Y. Drubin D. G., 2007 PtdIns(4,5)P 2turnover is required for multiple stages during clathrin- and actin-dependent endocytic internalization. J. Cell Biol. 177(2): 355–367. 10.1083/jcb.200611011 17452534 PMC2064142

[bib89] Sun Y. Leong N. T. Wong T. Drubin D. G., 2015 A Pan1/End3/Sla1 complex links Arp2/3-mediated actin assembly to sites of clathrin-mediated endocytosis. Mol. Biol. Cell 26(21): 3841–3856. 10.1091/mbc.E15-04-0252 26337384 PMC4626068

[bib90] Sun Y. Martin A. C. Drubin D. G., 2006 Endocytic internalization in budding yeast requires coordinated actin nucleation and myosin motor activity. Dev. Cell 11(1): 33–46. 10.1016/j.devcel.2006.05.008 16824951

[bib91] Suzuki R. Toshima J. Y. Toshima J., 2012 Regulation of clathrin coat assembly by Eps15 homology domain-mediated interactions during endocytosis. Mol. Biol. Cell 23(4): 687–700. 10.1091/mbc.E11-04-0380 22190739 PMC3279396

[bib92] Taylor M. J. Perrais D. Merrifield C. J., 2011 A high precision survey of the molecular dynamics of mammalian clathrin-mediated endocytosis. PLoS Biol. 9: e1000604 10.1371/journal.pbio.1000604 21445324 PMC3062526

[bib93] Tekletsadik Y. K. Sonn R. Osman M. A., 2012 A conserved role of IQGAP1 in regulating TOR complex 1. J. Cell Sci. 125(8): 2041–2052. 10.1242/jcs.098947 22328503 PMC3360921

[bib94] Ti S.-C. Jurgenson C. T. Nolen B. J. Pollard T. D., 2011 Structural and biochemical characterization of two binding sites for nucleation-promoting factor WASp-VCA on Arp2/3 complex. Proc. Natl. Acad. Sci. USA 108(33): E463–E471. 10.1073/pnas.1100125108 21676862 PMC3158158

[bib95] Urbanowski J. L. Piper R. C., 2001 Ubiquitin sorts proteins into the intralumenal degradative compartment of the late-endosome/vacuole. Traffic 2(9): 622–630. 10.1034/j.1600-0854.2001.20905.x 11555416

[bib96] Valdez-Taubas J. Pelham H. R. B., 2003 Slow Diffusion of Proteins in the Yeast Plasma Membrane Allows Polarity to Be Maintained by Endocytic Cycling. Curr. Biol. 13(18): 1636–1640. 10.1016/j.cub.2003.09.001 13678596

[bib97] Watanabe T. Wang S. Kaibuchi K., 2015 IQGAPs as Key Regulators of Actin-cytoskeleton Dynamics. Cell Struct. Funct. 40(2): 69–77. 10.1247/csf.15003 26051604

[bib98] Wendland B., 1999 Yeast epsins contain an essential N-terminal ENTH domain, bind clathrin and are required for endocytosis. EMBO J. 18(16): 4383–4393. 10.1093/emboj/18.16.4383 10449404 PMC1171513

[bib99] Wendland B., 2002 Epsins: adaptors in endocytosis? Nat. Rev. Mol. Cell Biol. 3(12): 971–977. 10.1038/nrm970 12461563

[bib100] Wendland B. McCaffery J. Xiao Q. Emr S. 1996 A novel fluorescence-activated cell sorter-based screen for yeast endocytosis mutants identifies a yeast homologue of mammalian eps15. J. Cell Biol. 135(6): 1485–1500. 10.1083/jcb.135.6.1485 8978817 PMC2133956

[bib101] Wesp A. Hicke L. Palecek J. Lombardi R. Aust T., 1997 End4p/Sla2p interacts with actin-associated proteins for endocytosis in Saccharomyces cerevisiae. Mol. Biol. Cell 8(11): 2291–2306. 10.1091/mbc.8.11.2291 9362070 PMC25709

[bib103] Winter D. Lechler T. Li R., 1999 Activation of the yeast Arp2/3 complex by Bee1p, a WASP-family protein. Curr. Biol. 9(9): 501–504. 10.1016/S0960-9822(99)80218-8 10322115

[bib104] Yang S. Cope M. J. Drubin D. G., 1999 Sla2p is associated with the yeast cortical actin cytoskeleton via redundant localization signals. Mol. Biol. Cell 10(7): 2265–2283. 10.1091/mbc.10.7.2265 10397764 PMC25442

